# Small steps for mankind: Modeling the emergence of cumulative culture from joint active inference communication

**DOI:** 10.3389/fnbot.2022.944986

**Published:** 2023-01-09

**Authors:** Natalie Kastel, Casper Hesp, K. Richard Ridderinkhof, Karl J. Friston

**Affiliations:** ^1^Amsterdam Brain and Cognition Centre, University of Amsterdam, Amsterdam, Netherlands; ^2^Institute for Advanced Study, University of Amsterdam, Amsterdam, Netherlands; ^3^Precision Psychiatry and Social Physiology Laboratory, Department of Psychiatry, CHU Sainte-Justine Research Center, Université de Montréal, Montreal, QC, Canada; ^4^Wellcome Centre for Human Neuroimaging, University College London, London, United Kingdom; ^5^Department of Developmental Psychology, University of Amsterdam, Amsterdam, Netherlands

**Keywords:** active inference, generalized synchrony, communication, social dynamics, cumulative culture, complex systems

## Abstract

Although the increase in the use of dynamical modeling in the literature on cultural evolution makes current models more mathematically sophisticated, these models have yet to be tested or validated. This paper provides a testable deep active inference formulation of social behavior and accompanying simulations of cumulative culture in two steps: First, we cast cultural transmission as a bi-directional process of communication that induces a generalized synchrony (operationalized as a particular convergence) between the belief states of interlocutors. Second, we cast social or cultural exchange as a process of active inference by equipping agents with the choice of who to engage in communication with. This induces trade-offs between confirmation of current beliefs and exploration of the social environment. We find that cumulative culture emerges from belief updating (i.e., active inference and learning) in the form of a joint minimization of uncertainty. The emergent cultural equilibria are characterized by a segregation into groups, whose belief systems are actively sustained by selective, uncertainty minimizing, dyadic exchanges. The nature of these equilibria depends sensitively on the precision afforded by various probabilistic mappings in each individual's generative model of their encultured niche.

## 1. Introduction

The study of cultural evolution examines how processes of transmission and selection at the individual level bring about population level patterns of cultural change. As a general overarching trend, models of cultural evolution have seen a steady increase in complexity, resulting from specialized theories from social psychology on the interconnected dynamics of culture. For instance, a recent model of cultural systems (Jansson et al., 2021) applied a framework that implements the structural dependencies between cultural traits and the emergent ways that these dependencies influence the acquisition of cultural transmission.

Another step in the direction of increasing the complexity and systems view of culture has been the investigation of the relationship between population structure and the capacity for a culture to accumulate beneficial cultural traits over time (i.e., cultural accumulation), which has been a particular topic of interest in the past decade. Although some empirical tests provide support for the hypothesis that effective population size constraints cumulative cultural evolution (Derex and Mesoudi, 2020), there is contradictory evidence regarding the relationship between population size and cultural accumulation (Kempe and Mesoudi, 2014). Another study theorizing about the foundations for the uniquely human capacity for cultural accumulation suggests that this capacity is rooted in a unique foraging niche which can still be observed in few hunter-gatherer populations. This niche, encompassing social interactions such as cooperation with unrelated individuals and social division of labor, underlies a task specialization which spreads cultural knowledge across individuals. This task division, a multilevel social structure which evolved as an adaptation to the environment, may explain human collective intelligence and its unique capacity for sophisticated cumulative culture (Migliano and Vinicius, 2022).

While the increase in the use of dynamical modeling in the literature on cultural evolution makes current models more mathematically sophisticated, these models have yet to be tested or validated (Kashima et al., 2017). Review of the literature reveals that one line of research that has been especially fruitful in that it can be validated experimentally are Bayesian models that create detailed models of cognition and have had remarkable success in producing predictions qualitatively in accord with experimental results (Kempe and Mesoudi, 2014). Currently these models have only been applied to relatively low level cognitive processes, but the creation of high level cognitive maps at the individual level as well as modeling the emergence of cultural change on the population-wide dynamics represents a promising line of future work.

The dynamics underlying the evolution of culture consist of three processes that are typically studied separately: the introduction of novel beliefs and practices to a culture (i.e., innovation), the transmission of established beliefs and practices within a population (i.e., innovation diffusion), and changes in their prevalence (Kashima et al., 2019). The term “cultural transmission” typically denotes the transference and spread of any particular fashion, ideology, preference, language, or behavior within a culture (Creanza et al., 2017). A prominent stream of quantitative models for cultural transmission are inspired by epidemiology, and convert models used for predicting the spread of a virus to formalize the spread of an idea (Bettencourt et al., 2006).

While the comparison of an idea to a (non-mutating) virus has its benefits from a formal perspective, it implies the controversial notion that an idea is simply copied during its transmission through cultural exchange between individuals. This notion is not only intuitively insufficient for a realistic characterization of communication dynamics, but also conflicts with established theoretical models of transmission on these same grounds.

Current literature in cultural psychology indicates that rather than being simply duplicated during transmission, cultural beliefs and practices are modified through the active interpretation of each individual (Kashima et al., 2019). Furthermore, psychological research indicates that effective human communication can be characterized by (usually only partially) common realities in which conversation partners share an intersubjective reference frame (Clark and Brennan, 1991; Echterhoff et al., 2009). Accordingly, conversation partners have to be understood as active participants that co-create these partially shared reference frames in a self-organizing fashion over the course of each interaction. Intersubjective theories of communication aim to account for those underlying dynamics that—slightly paradoxically—both enable and (to some extent) require the co-creation of (partially) shared reference frames amongst interlocutors. In contrast, traditional formulations have tended to oversimplify communication in terms of back-and-forth exchanges based on (largely) fixed symbolic meaning systems, implicitly presupposing those shared reference frames in an *ad-hoc* manner.

The theory of cumulative culture (Stout and Hecht, 2017; Dunstone and Caldwell, 2018) expands on the notion that progressive alterations of cultural beliefs and practices are intrinsic to all cultural exchanges because they are embodied, expressed, and interpreted differently by each individual participant of the ensemble (Dean et al., 2014). While efficient cultural exchanges do tend to be grounded in similar physical substrates across and within individuals (e.g., facial expressions, body language, etc.), the high abstraction levels and malleability of these substrates render cultural dynamics in a different class than phenomena that are wholly dependent on consistency across genetic substrates, such as sexual reproduction and disease spread. Of course, genetics does play a crucial role in the range of phenomena associated with gene-culture co-evolution, but that will be explored in future work.

Although there appears to be some degree of consensus on the intrinsic complexity of culture, it remains an outstanding challenge to provide convincing quantitative accounts of its full glory (Buskell et al., 2019). This article aims to act as a stepping stone toward tackling this challenge of characterizing cumulative culture by developing a multi-agent model based on deep active inference account. This work was developed by reformulating and greatly expanding upon a much shorter conference contribution that was previously published by the two lead authors (Kastel and Hesp, 2021) and publicly available as an open-source preprint. The three Figures that were adapted or reprinted from that conference paper have been highlighted (**Figures 2**, **8**, **9**). Textual overlap has been minimized and reprinted Figures have been highlighted where relevant.

## 2. Methods

An emerging conclusion from the literature is that the term “transmission” for describing the spread of cultural information seems impoverished, as it leaves out the retention of cultural information. As implied by active inference—and theoretical models of communication—the acquisition of cultural beliefs is as fundamental to the understanding of cultural information spread as their transmission. For this reason, we will henceforth be referring to what is known in the literature on cultural transmission as communication, or more technically: the local dynamics of cumulative culture. Although we will use the term “communication” and “transmission” interchangeably in this paper, It is important to note that communication does not always imply cultural transmission. Cultural transmission, also known as cultural learning, refers to the learning of social behaviors that occurs in every new generation in a particular society (Nicol, 1995). Since cultural transmission only occurs when social behaviors or beliefs are learned, communication only truly implies transmission when what is being communicated has been picked up and solidified in the receiver's cognitive model. We assume this kind of communication in our simulations, which is why under our account communication does imply transmission.

Conversely, cultural transmission can occur without communicating information through language. It has been suggested that humans learn the social behaviors of their culture through immersive participation in cultural practices that selectively shape attention and behavior. This is a form of implicit learning, where agents infer other agents' expectations about the world and how to behave in a social context. It is even argued that implicit learning of information about other people's expectations constitutes the primary domain of statistical regularities that humans leverage to predict and organize behavior (Veissière et al., 2020). Although cultural transmission does not necessarily require verbal communication, we assume this kind of communication in our simulations.

### 2.1. Simulating the local dynamics of communication

In our model, cultural transmission is cast as the mutual attunement of actively inferring agents to each other's internal belief states. This builds on a recent formalization of communication as active inference (Friston and Frith, 2015) which resolves the problem of hermeneutics, (i.e., provides a model for the way in which people are able to understand each other precisely, despite lacking direct access to each other's internal representations of meaning) by appealing to the notion of generalized synchrony as signaling the emergence of a shared narrative to which both interlocutors refer. From the perspective of active inference, agents of a socio-cultural system infer the belief states of those in their environment and update their own representations accordingly. An emergent property of this bi-directional inference—and implicit belief updating—is the synchronization of belief states among the cultural ensemble (Palacios et al., 2019).

In nature, generalized synchrony emerges from a specific coupling between the internal states of dissipative chaotic systems (Pikovsky et al., 2003). As a fundamental concept in complex systems theory closely related to stochastic resonance (Nicolis and Nicolis, 2012), it is typical of complex nonlinear dynamics that afford the coexistence of chaotic and ordered subsystems (also called chimera patterns; see, e.g., Zakharova, 2020; Haugland, 2021). In active inference, the coupling of agents' internal states is made possible through communication, as it allows interlocutors to mutually influence each other and enter into a bidirectional action-perception cycle that can be described as coupled dynamical systems (Friston and Frith, 2015; Constant et al., 2018). When active inference agents engage in the coupled dynamics of communication, generalized synchrony between their internal states emerges from their mutual efforts to minimize uncertainty—as scored mathematically with (variational) free energy. Put simply, generalized synchrony ensures the greatest mutual predictability error resolves the greatest amount of collective uncertainty.

Our model of communication builds on the notion of generalized synchrony to suggest that the emergence of synchrony from the coupled communication of active inference agents may be operationalized as a particular convergence between their respective generative models. That is, when we simulate the belief-updating dynamics of communicating agents, the cultural reproduction of a particular idea takes the form of a learnable convergence between their respective belief states (expressed as generative models) and distinct representations combine into one synchronized, shared model of the world.

**Figure 1:**
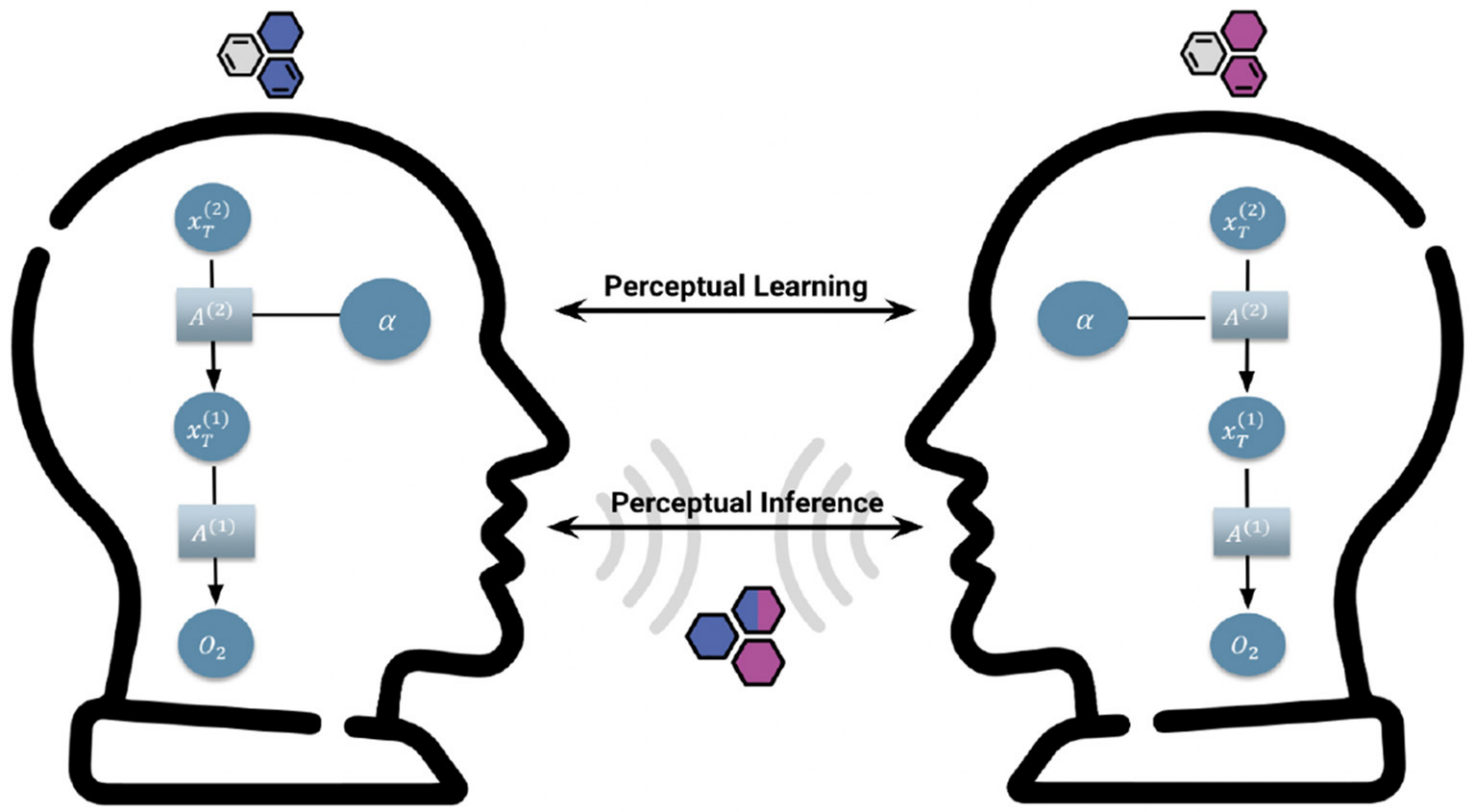
Communication Coupling Parameters. Our model defines two groups of parameters that couple the internal states of agents: Learning and inference. Perceptual learning (A2) is the learning of associations between emotional valence and belief states that guide the long term actions of our agents who hold and express beliefs. This learning happens at slow time scales, accumulating across multiple interactions and used to modify models over extended periods of exchange. Perceptual Inference (A1)—namely, sensitivity to model evidence—operates on fast time scales and is direct and explicit to agents during dialogue. Importantly, we hypothesized that without precise evidence accumulation, agents would be insensitive to evidence regarding the belief state of the other, and their internal states would not converge.

Formally, our model defines perceptual inference in terms of a coupling parameter linking the internal states of interlocutors through dialogue (Figure 1). Also understood as sensitivity to model evidence (A1), perceptual inference is a direct and explicit form of coupling that occurs over the span of a single dialogue such that it is hypothesized to modulate agents' convergence of internal belief states during communication.

While throughout the narrative of this paper we have characterized the alternative “idea” as having actual content, we have intentionally left it unspecified such that it could also be taken to simply refer to a blanket disagreement with the ideas or practices representing the “status quo.” In that sense, it is consistent with simulation work on social dynamics suggesting that cultural extremism can arise without the formulation of alternatives (e.g., see Kashima et al., 2021).

### 2.2. Simulating the global dynamics of cumulative culture

Cultural beliefs and practices spread within a society through communication, a process which we have referred to as the local dynamics of cumulative culture. This description is appropriate because the accumulated outcomes of each (local) dyadic interaction collectively determine the degree to which an idea is prevalent in a culture. Moving from local communication dynamics to the prevalence of a communicable idea—in a cumulative culture—is what we will refer to as the global dynamics of cumulative culture.

In our simulations of a cumulative culture, 50 active inference agents simultaneously engage in local dyadic communication as illustrated. Forty-nine of our 50 agents were initialized as adhering to a similar idea, which could be regarded as the status quo (indicated with the color red later on), while the initial strength of their adherence to this consensus varied across individuals because we generated the parameters of their generative models from various probability distributions to characterize variability in the population (described in the Appendix). The same holds for other modulators of cognitive, affective, and behavioral variability, such as (1) expectations about each other's expressions, (2) habit formation, and (3) emotional valence states (all described below). Jointly, the emergent effects of these individual differences gave rise to factions that vary in their adherence to the current consensus, in a way reminiscent of political diversity in real-life cultural environments: strict conservatives, centrists, and skeptics (see also **Figure 6** below). In order to illustrate this spectrum, we introduced one agent (labeled “rogue agent” in **Figure 6**) whose idea strongly contradicted the consensus and who was fully resistant to the consensus idea. When we introduced this agent adhering to a divergent idea to the population, it propagated *via* pseudo-random engagements of agents in dialogue. In this simulated world of actively inferring agents, their individual mental (generative) models were slightly modified with every interlocutor they encounter, as their distinct representations converged to a shared narrative (Friston and Frith, 2015; Constant et al., 2019). The attunement of interlocutors to each other's generative models on the (local) microscale thus translated over time and with multiple encounters into collective free energy minimization on the (global) macroscale.

## 3. A generative model of communication

For a formal (variational free energy) proof of principle, we offer an active inference account of cultural dynamics. A foundational step in this endeavor is the formulation of generative models underlying the decision making of agents that can be deployed in simulations.

Active inference assumes that the brain-body system mimics a Bayesian inference machine by embodying a model of itself acting in the world and using local observations to secure evidence for that model. This model is “generative” in that it generates predictions of what observational data should look like, given that the model is correct. Predictions are compared with actual observations and any discrepancies (known as “prediction errors”) are used to update the generative model (Smith et al., 2019). This (Bayesian belief) updating can be at a fast timescale corresponding to inference about the hidden causes of observations—or at a slow timescale corresponding to learning the parameters of the generative model, which best explain the inferred causes. For an elaboration on the mathematical foundations of active inference, the reader is referred to Friston K. et al. (2017).

In our simulations, agents attempt to convince each other of a cultural belief under generative models that operate with local information only. We formulate these generative models as a partially observed Markov decision process (MDP), where beliefs take the form of discrete probability distributions (for technical details on MDPs in active inference, see Hesp et al., 2019). To simulate active inference under these models, one specifies variables—such as hidden states (*x*, *s*), observable outcomes (*o*) and one-step action policies (*u*)—alongside parameters specifying the probabilistic relationships between the variables in question.

Agents' recollection of a visit is thus an expression of humans' innate ability to infer each other's expectations, which makes human cognition, sociality, and culture possible at all (Veissière et al., 2020). This rests on the idea that humans, having evolved to rely on elaborate and highly coordinated action, have expectations regarding other agents' sharing aspects of their own generative model, and thereby believing that other agents have those expectations as well. These carefully and implicitly coordinated and co-constructed expectations allow agents from a particular culture to learn what to expect from each other and leverage those expectations to act accordingly in their environment. In our model these expectations are manifested as agents' information and preference-seeking, which are biased toward the selection of similar interlocutors to engage with, in conversation.

**Figure 2:**
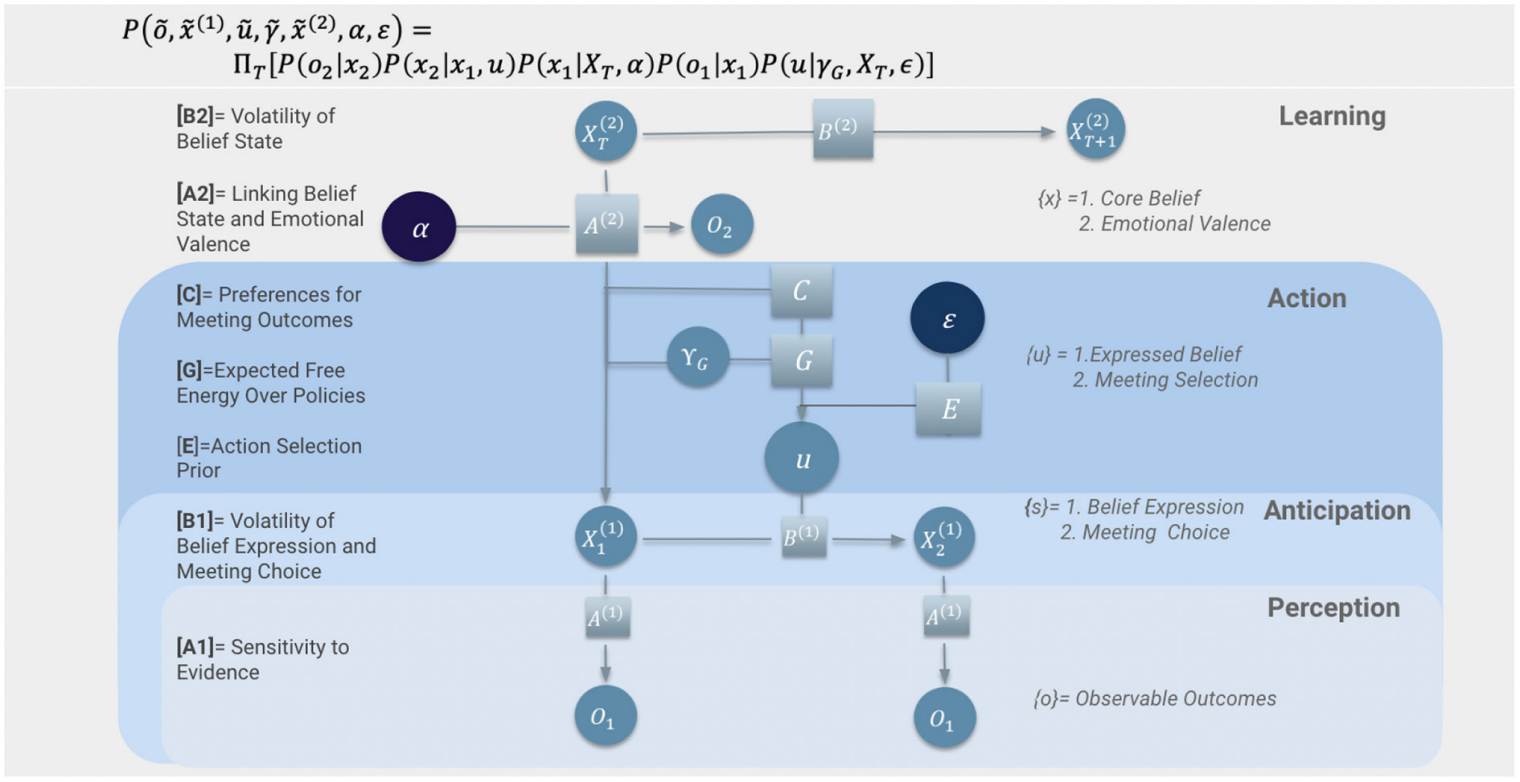
A generative model of communication. This Figure was reprinted from an open-source preprint of a conference paper, with permission of the authors (Figure 1 of Kastel and Hesp, 2021). Variables are depicted as circles, parameters as squares and concentration parameters as dark blue circles. Visualized on a horizontal line from left to right, states evolve in time. Visualized on a vertical line from bottom to top, parameters underwrite a hierarchical structure that corresponds to levels of cognitive processing. Parameters are listed on the left of the generative model and variables are on the right.

MDPs allow for the construction of a deep hierarchical model comprising nested levels of complexity. Below we will describe those levels and detail the cognitive processes that take place within each one (Figure 2).

**Figure 3:**
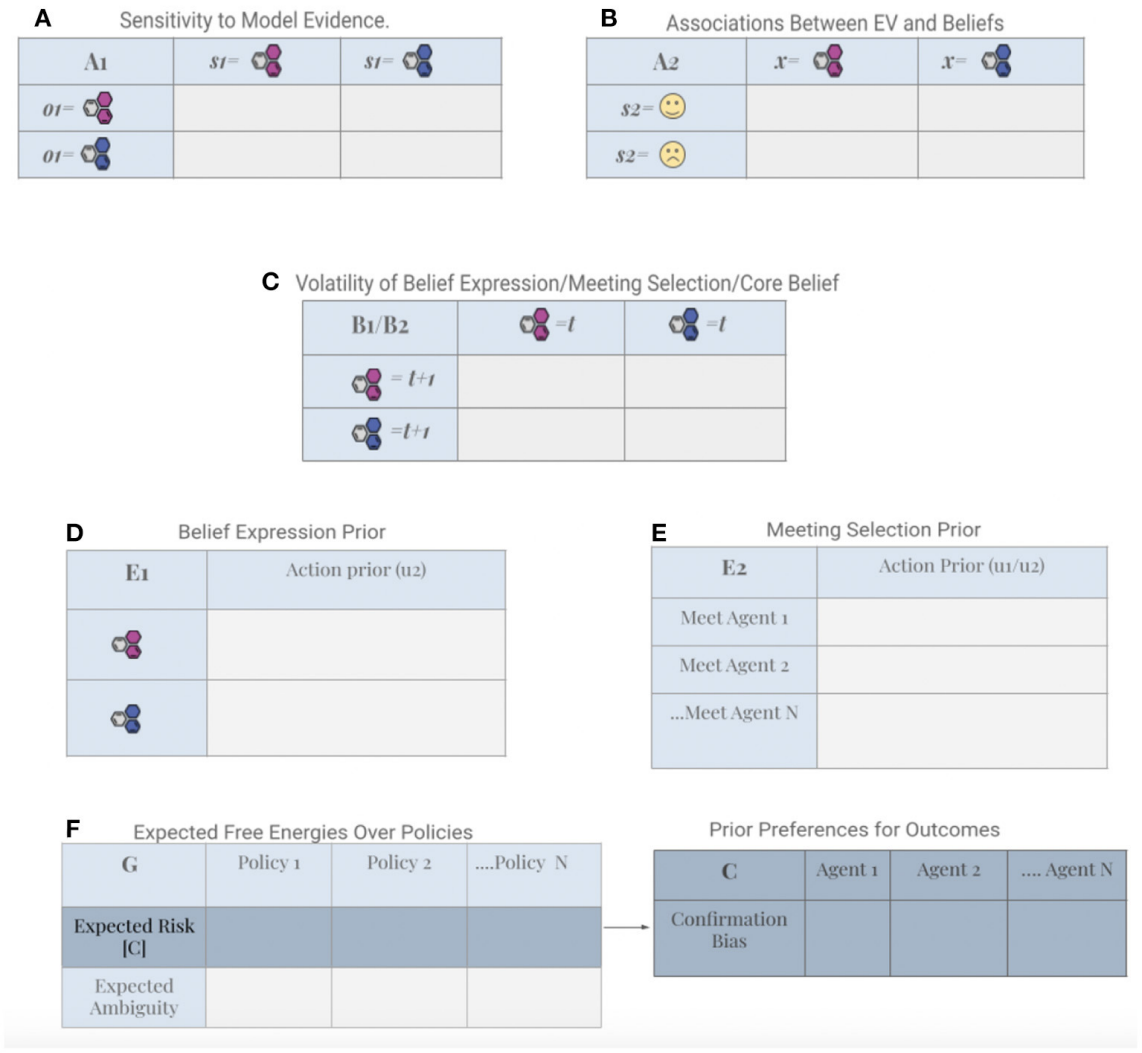
Generative model parameters. **(A)** The *A*1 matrix specifies an agents' perception of an interlocutors' expressed beliefs. The precision of this likelihood mapping determines the agent's sensitivity to these expressions. **(B)** The *A*2 matrix represents what the agent has learned about the mapping between her high and low level beliefs. **(C)** The *B* matrix, or state transition probabilities, represent what the agent has learned about how hidden states evolve over time. The precision of B matrices can be understood as encoding the volatility of belief states. **(D)** The *E*1 matrix is one of two habitual contributions to action selection. It covers two possible outcomes for expressing beliefs. This contribution is specified on a continuous range between (0,1), where the extremes correspond to either complete confidence in denying or supporting the claim. **(E)** The *E*2 matrix is the second habitual component for action, and it holds 50 possible outcomes for meeting selection (i.e., the probability for meeting each agent in the population). **(F)** The expected free energy of allowable policies (i.e., choices or actions) is indicated with *G*, which entails two components: 1. expected risk (the KL-divergence from the *C* matrix and biasing toward confirming one's preferred ideas) and expected ambiguity, which biases toward meeting new agents with unknown beliefs. Note: The purpose of this Figure is to draw the attention of the reader to the general form of the matrices shared across the simulated agents. The tables are left empty because, for any individual agent in the simulated population, each of these objects contains specific numbers, which are initially generated procedurally from various probability distributions (described in the text) and change throughout the simulation as the agents interact in their shared environment. Specific numbers could at best describe only one particular agent at a given instance of time (which does not represent the entire population). Furthermore, the probability distributions used to generate initial values do not reflect the additional steps required such as, e.g., the renormalization procedures involved in applying a softmax operator. Finally, it would also occlude the fact that certain entries (e.g., the expected free energy) will vary over time during a simulation.

For our simulations, six kinds of matrices were parameterized (*A*, *B*, *C*, *E*, *C*, and *G*) using two kinds of concentration parameters (α,ε) for Dirichlet distributions, and temperature and rate parameters for precision terms (indicated with γ; see Figure 3 and Appendix A9).

### 3.1. Perceptual inference

The first level of this generative model captures how agents process belief claims they are introduced to through conversation with other agents. The perception of another's beliefs requires prior beliefs (represented as likelihood mapping *A*1) about how hidden states (*s*1) generate sensory outcomes (*o*). Specifically, our agents form expectations about the likelihood of encountering expressions of particular ideas, given their beliefs about the degree of consensus in the agent's social circles and their past experiences with individual agents. Parameterizing this (likelihood) mapping in terms of precision can be understood as parameterizing each agents' sensitivity to the claims of others. High precision here corresponds with high sensitivity to claims. The likelihood precisions for each agent were generated from a continuous gamma distribution, which was skewed in favor of high sensitivity to evidence at the population level (see Figure 2: Perception).

#### 3.1.1. Perceptual inference as a coupling parameter

The sensitivity to another agent's claims (*A*1), represents the explicit coupling between interlocutors, in terms of how much belief updating one agent can induce in another agent. It is a key element in our simulations (Figure 1). We call this parameter explicit because it modulates the direct (i.e., explicitly articulated) and immediate (i.e., occurs over the course of a single interaction with an agent) influence of agents' claims on the beliefs of others (Friston and Frith, 2015). In other words, sensitivity to claims—encoded by the likelihood precision—couples the belief states of interlocutors *via* their claims or utterances to each other (**Figure 8**). Crucially, belief updating depends not only on their adherence to each other's claims but also a certain (varying) degree of commitment to their own beliefs. The balance is determined by each agents' sensitivity to sensory evidence; i.e., the claims of interlocutors.

Technically, we can describe belief updating in terms of the generative model in Figure 3 as follows:

- Initial higher-level core support for the idea at the beginning of the simulation (*T* = 1):

(1) $$\begin{array}{l} P\left(x_{core,T=1}^{\left(2\right)}\right)=\text{Cat}\left({\text{D}}^{\left(2\right)}\right) \end{array}$$

- Evolving higher-level beliefs after each meeting (*T* > 1), introducing volatility over time:

(2) $$\begin{array}{l} P\left(x_{core,T}^{\left(2\right)}|x_{core,T-1}^{\left(2\right)}\right)=\text{Cat}\left({\text{B}}^{\left(2\right)}\right) \end{array}$$

- Initializing lower-level beliefs about the claims of others, based on higher-level (cross-meeting) beliefs:

(3) $$\begin{array}{l} P\left(x_{idea}^{\left(1\right)}\right)=\text{Cat}\left({\text{x}}_{core,T}^{\left(2\right)}\right) \end{array}$$

- Updating beliefs about the other agent's belief based on their claims (Appendix A7), within the current meeting:

(4) $$\begin{array}{l} Q\left(x_{idea}^{\left(2\right)}\right)=Q\left(x_{idea}^{\left(1\right)}\right)=\text{Cat}\left({\text{o}}_{expr}\right) \end{array}$$

- Updating of core belief based on claims of self and another agent after each meeting (detailed descriptions of the computations involved in this belief updating can be found in the Appendix):

(5) $$\begin{array}{l} Q(x_{core}^{\left(2\right)})=\sigma (\ln\;x_{core}^{\left(2\right)}+\gamma_{A,self}^{\left(2\right)}\ln\;o_{expr,self}\;\\ \;+\gamma_{A,other}^{\left(2\right)}\ln\;o_{expr,other}) \end{array}$$

### 3.2. Anticipation

At the first level, our generative model specifies the agents' beliefs about how hidden states (detailed in Appendix A2) evolve over time. The precision of state transition probabilities in B1 (Figure 3C) specifies the volatility of an agent's meeting location (*s*2) and beliefs in particular claims (*s*1) [*B*1]. For each agent, this precision parameter is sampled from a gamma distribution, determining the a priori probability of changing state, relative to maintaining a current state. Note that belief states themselves are defined on the continuous range (0, 1) (i.e., as a probability distribution on a binary state), such that repeated state transitions tends to result in a continuous decay of confidence over time, in the absence of new evidence (where the rate of decay is inversely proportional to the precision of *B*1) (see Figure 2: Anticipation).

### 3.3. Action

After inferring and anticipating hidden states, our agents conduct deliberate actions to minimize expected free energy (the generative model for action is detailed in Appendixes A4, A5). At each time point, a policy (*u*) is chosen out of a set of possible action sequences. In our simulations, two types of actions are allowed: selecting an agent to meet at each given time point (*u*2) and selecting a specific claim to express in conversation (*u*1). The first allowable action covers 50 possible outcomes (one for each agent in the simulation) while the second corresponds to denying or supporting the claim. In order to simulate variability in an agents' confidence in a belief claim, the claim is generated for each conversation from a beta distribution that is parameterized by the speaker's (phenotype-congruent) action model. Each policy under the *G* matrix (Figure 3F) specifies a particular combination of actions, and the policy that minimizes expected free energy is chosen (see Figure 2: Action).

#### 3.3.1. Habitual belief expression and meeting selection

At the low level of cognitive control, each agent starts with a baseline prior expectation concerning the probability of a particular policy being selected (action prior probabilities [*E*1 and *E*2], Figures 3D,E). This parameter can be understood as modeling a habitual cognitive process, where an agent's current one-step policy (*u*) is biased toward previously selected actions (*u*1, *u*2). In our simulations, agents observe and track previous actions *via* the accumulation of a concentration parameter (ε), thus enabling continued updates to action priors, which can either strengthen or weaken previous habits (details for habit learning of belief expression and meeting selection are provided in Appendix A8).

#### 3.3.2. Voluntary belief expression

At the high level of cognitive control, agents incorporate a series of processes underlying the selection of a particular claim for expression (*u*2). In addition to habitual factor (E), this selection involves several considerations. First, an agent considers their core belief state (*x*), and the way this state a priori maps on to one of two discrete emotional valence states (*s*2) *via* a likelihood mapping [*A*2] (Figure 3B). Emotional Valence (*EV*) is defined as the extent to which an emotion is positive or negative (Russell and Barrett, 1999), such that agents' core beliefs are a priori associated with either positive emotional valence or negative emotional valence (with some probability). As a minimal form of vicarious learning, the initial mapping is further updated based on associations agents observe between their interlocutors' expressed claims and *EV*-value (details of the generative process underlying belief expression and emotional valence are provided in Appendix A6). The initial mapping therefore involves minimal precision for the expected *EV* for the alternative belief since agents are first introduced to this belief (and associated *EV*) during the simulations. For this reason, the initial likelihood mapping between states is updated throughout our simulation *via* a crucial concentration parameter (α) which will be elaborated on under level 4.

The inferred *EV* state is then used to generate an action precision (γ) such that positive *EV* generates high confidence in action selection (*u*1) and negative *EV* generates low confidence. Higher confidence values produce higher precision on the expected free energy (*G*) for one's belief claim expressed in the current conversation.

EV states are generated from core belief states, using a (learnable) likelihood mapping:


(6) $$\begin{array}{l} P\left(x_{sat}^{\left(1\right)}|x_{core}^{\left(2\right)}\right)=Cat\left({\text{A}}_{sat}^{\left(2\right)}\right) \end{array}$$

Confidence of belief expression is generated using a Gamma distribution, where the rate parameter β_*expr*_ is the Bayesian model average of β^(+, −)^ values associated with high and low satisfaction:


(7) $$\begin{array}{l} P\left(\gamma_{expr}\right)\approx \text{Γ}\left(1,\beta_{expr}\right) \end{array}$$

(8) $$\begin{array}{l} \beta_{expr}=\beta^{\left(+,-\right)}\cdot \;{\text{x}}_{sat}^{\left(1\right)} \end{array}$$

where β^(+, −)^ = [0.25;2.0]

The expression of beliefs is guided by current core beliefs (scaled with satisfaction-dependent γ_*expr*_) and by habitual belief expression **E**_*expr*_ (scaled with a fixed parameter γ_*E,expr*_):


(9) $$\begin{array}{l} P\left(u_{expr}|\gamma_{expr}\right)=\sigma \left(-\gamma_{expr}ln\;{\text{x}}_{core}^{\left(2\right)}\;+\gamma_{E,expr}{\text{E}}_{expr}\right) \end{array}$$

The intrinsically stochastic and itinerant nature of the generative process of communication is modeled by using a two-dimensional Dirichlet distribution to generate observed expressions on the range (0,1), where each agent's belief expression prior *P*(*u*_*expr*_|γ_*expr*_) is used to specify their concentration parameters (multiplied by 12 to reduce variance):


(10) $$\begin{array}{l} {\text{o}}_{expr}=\text{Dir}\left(12{\text{u}}_{expr}\right) \end{array}$$

#### 3.3.3. Voluntary meeting selection

**Figure 4:**
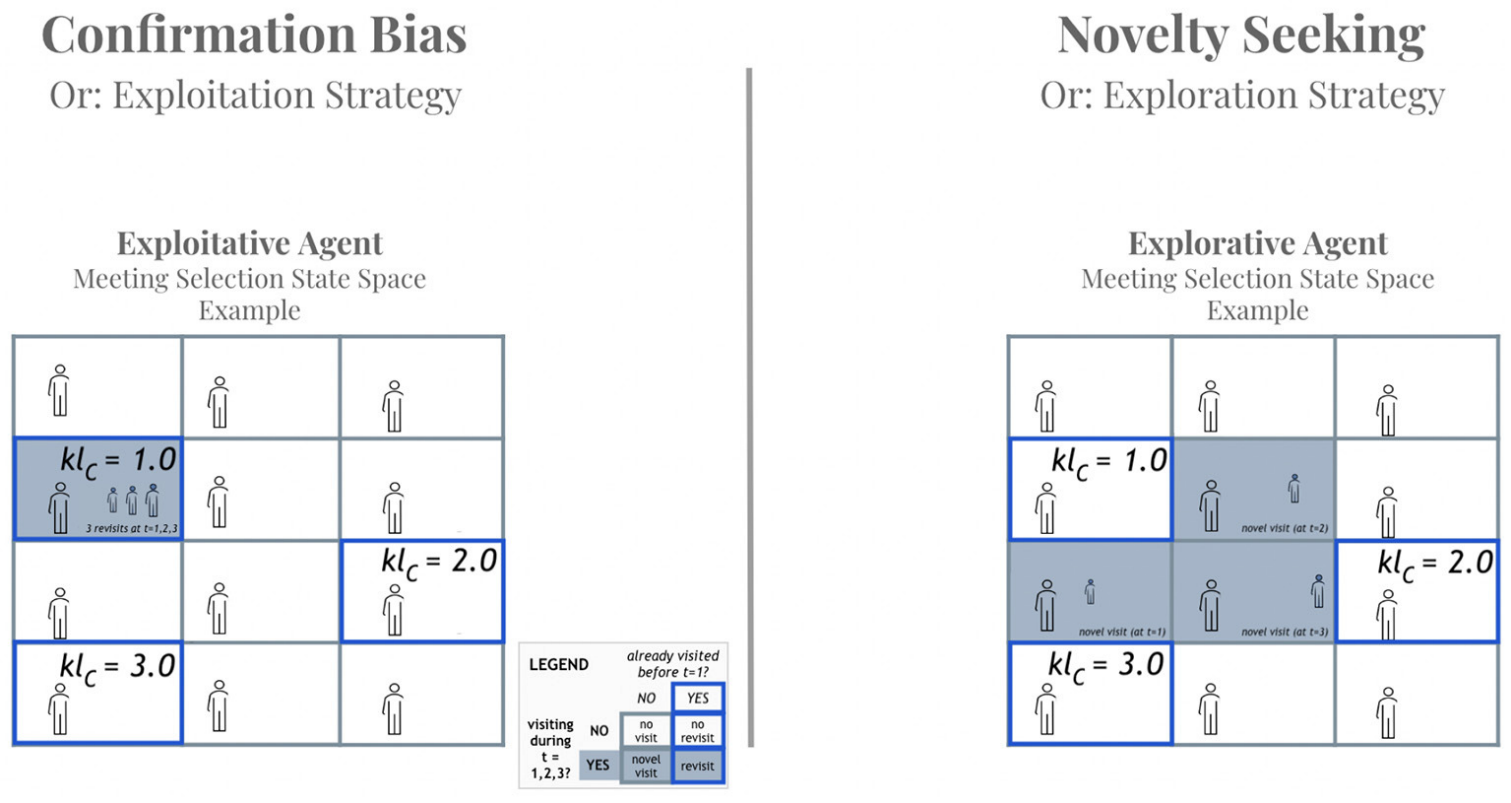
A simplified example of exploitation- and exploration-driven strategies for meeting selection. This Figure illustrates the behavioral differences between the extreme cases of being fully driven by exploitation **(Left)** or exploration **(Right)**. Each cell on the grid corresponds to a potential interlocutor for these agents, who make decisions in three consecutive time steps (*t* = 1, 2, 3) and have previously engaged with three other interlocutors (marked with blue rectangles), where we use the shorthand *kl*_*C*_ to indicate the pragmatic component of the expected free energy G_*pragmatic,visit*_ = o_*expr,visit*_ · (ln o_*expr,visit*_ − C_*idea*_), which corresponds to the KL divergence between expectations about the interlocutor at that location (as informed by previous visits) and the preferred ideas of our agents, such that lower values correspond to a better match. Cells that are visited during *t* = 1, 2, 3 are filled with granite. The exploitation-driven agent **(Left)** simply revisits three times a known interlocutor with the lowest *KL*_*C*_. In contrast, the exploration-driven agent **(Right)** prefers novel visits and switches to an unknown agent every time step. In the simulations presented later, agents will dynamically balance these two strategies as their preferences themselves evolve over time.

While the choice of interlocutor is predetermined in a dyad, our multi-agent simulations required a specification of the process behind agents' selection of a conversational partner (*s*3) at each of the (hundred) time points. Building on previous work on active inference navigation and planning (Kaplan and Friston, 2018), meeting selection in our model is represented as a preferred location on a grid, where each cell on the grid represents an agent to meet (Figure 4).

Importantly, agents differ in their action model of which agent to visit at each time point. Their individual choices are guided by expected free energy G (Figure 3F) which entails maximizing the expected utility of an action (known as pragmatic value) as well as maximizing the expected information gain (known as epistemic value). These two values constrain each other such that maximizing both simultaneously is partially (but not entirely) paradoxical (as illustrated in Figure 4). These constraints may also be understood as formalizing the exploration-exploitation trade-off, where epistemic value (exploration) refers to the benefit of searching to get a better estimation of promising areas that offer pragmatic value (exploitation) (Friston and Frith, 2015).

Mathematically, action selection was formalized as follows:


(11) $$\begin{array}{l} P\left(u_{visit}|\gamma_{G,visit}\right)=\sigma \left(-\gamma_{G,visit}{\text{G}}_{visit}+\gamma_{E,visit}{\text{E}}_{visit}\right) \end{array}$$

Here, **G**_*visit*_ represents the expected free energy:


(12) $$\begin{array}{l} {\text{G}}_{visit}={\text{o}}_{expr,visit}\cdot \left(\ln\;{\text{o}}_{expr,visit}-{\text{C}}_{idea}\right)+\text{H}\cdot \;{\text{x}}_{idea,visit} \end{array}$$

An agent traveling to visit:


(13) $$\begin{array}{l} x_{idea,visit}^{\left(1\right)}={\text{B}}_{visit}^{\left(1\right)}x_{idea,home}^{\left(1\right)} \end{array}$$

Expectations about the support for an idea expressed by each potential agent one could visit:


(14) $$\begin{array}{l} {\text{o}}_{expr,idea}={\text{A}}_{idea}^{\left(1\right)}{\text{x}}_{idea,visit}^{\left(1\right)} \end{array}$$

Individual preferences about the support for the idea:


(15) $$\begin{array}{l} {\text{C}}_{idea}=\ln\;\left({\text{A}}_{C}^{\left(2\right)}{\text{x}}_{core}^{\left(2\right)}\right) \end{array}$$

Finally, expectations about a potential reduction in ambiguity about the support for an idea by a particular agent reflects one's recollection about their most recent visit to this other agent. *H*_*j*_ = 0 if an agent can remember a recent visit (i.e., there is no ambiguity left to reduce), and 0.1 otherwise.

Crucially, both types of (information and goal seeking) preferences are absorbed into expected free energy. Pragmatic value translates into a bias toward meeting agents with similar beliefs at a given time point. This bias reflects the widely observed phenomenon in psychology research that people's choices tend to be biased toward confirming their current beliefs (Nickerson, 1998). Confirmation bias, or a state-dependent preference (*C*) for meeting “belief compatible” agents, biases action selection through the risk component of expected free energy (*G*) (Figure 3F). Under active inference, a preference for meeting agents with similar beliefs increases the propensity for generalized synchronization, which underwrites the emergence of (expected free energy reducing) shared expectations (Hesp et al., 2019).

In contrast, emphasizing epistemic value translates into a bias toward meeting agents whose beliefs are unknown at a given time point. This bias reflects the extent to which agents are driven by the minimization of the ambiguity component of expected free energy (*G*; Figure 3F) about the beliefs of other agents. Novelty seeking, or a proclivity for encountering novel agents with unknown beliefs is a strategy for maximizing information gain. Also understood as intrinsically motivated curiosity behavior (Friston K. J. et al., 2017), maximization of epistemic value helps individuals to better predict the consequences of their actions (e.g., when they decide which agent to meet) as they reduce uncertainty about hidden states of their environment, whether real or imagined (e.g., in this case it refers to the ideas supported by other agents).

Because of our method of procedural generation of various (hyper)parameters from various probability distributions (described in more detail in the Appendix), a continuous spectrum of cognitive, behavioral, and affective tendencies emerged in our simulations due to the large variety of possible combinations. In principle one could obtain any range of behaviors from this method of procedural generation based on a set of probability distributions for all these hyperparameters, which could hence be fitted to population data. A full-blown analysis of all the emergent variability in the simulated populations is beyond the scope of the current paper.

**Figure 5:**
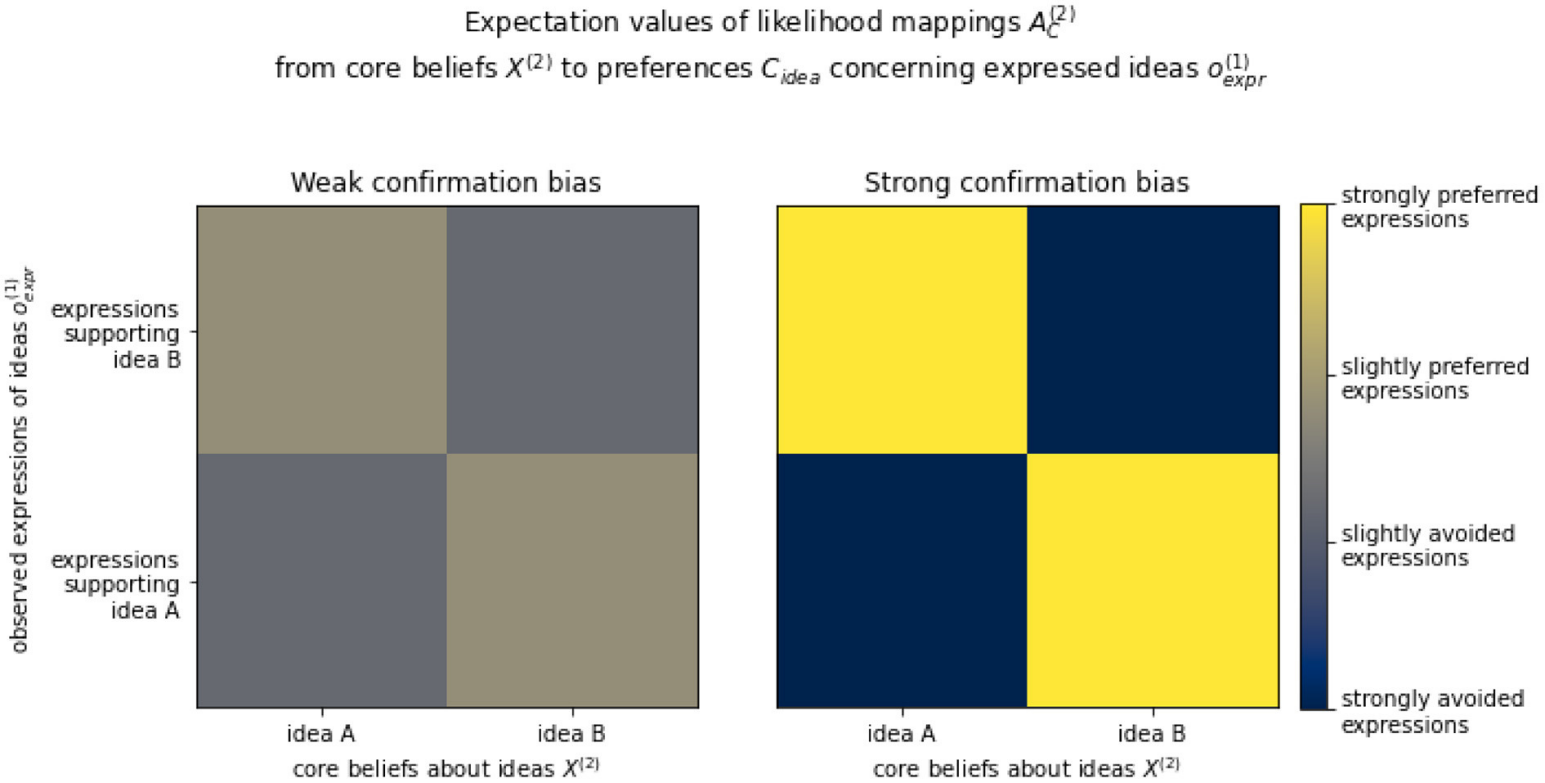
Two sets of expectation values of the Dirichlet distributions used to generate top-down likelihood mappings $A_{C}^{\left(2\right)}$, from core beliefs about ideas *X*^(2)^ to preferences concerning expressed ideas *C*_*idea*_, representing the two distinct populations for which parameters were initialized with different degrees of confirmation bias. Weak confirmation bias **(Left)** corresponded to mild preferences for observed expressions to confirm core beliefs, while strong confirmation bias **(Right)** corresponded to a strong preference for observed expressions to confirm core beliefs (essentially a one-to-one mapping).

For the sake of our demonstration, a clear distinction between agents with high and low confirmation bias was introduced in our simulations by drawing individualized hyperparameters from two distinct sets of Dirichlet distributions (illustrated in Figure 5; described in the Appendix) to obtain each agent's likelihood mapping from higher-level core beliefs to lower-level preferences concerning observed expressions. The resulting distinct populations could have emerged from, e.g., cultural segregation where different cultural subgroups have developed different priorities in guiding social interactions—in this case guided more or less strongly by confirmation of core beliefs.

**Figure 6:**
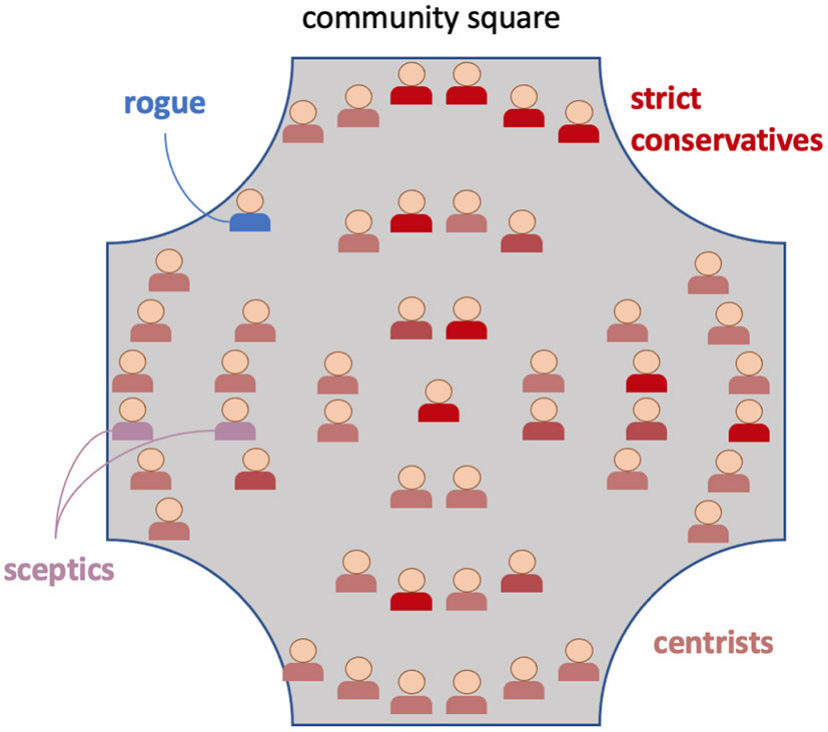
A depiction of the community square with its initial opinion distribution: One idea (red) was supported by almost all agents, with some variation due to individualized model parameters, roughly dividing into “strict conservatives,” “centrists,” and a few “skeptics.” At first, the alternative idea (blue) is supported by only one, stubborn agent (“rogue”).

Novelty-seeking tendencies were not explicitly coded and simply emerged from the parameters that regulate the relative impact of epistemic vs. pragmatic value in the expected free energy, although it should be clear that high confirmation bias tends to suppress novelty-seeking. In Figure 6, the distinction between “strict conservatives,” “centrists,” and “skeptics” was used to qualitatively describe the emergent continuous spectrum purely for communicative purposes and should not be taken as a definite discretization.

### 3.4. Perceptual learning

On this level of belief updating, agents learn contingencies; for example, how core belief states (specified in Appendix A1) change over time (*B*2) (Figure 3C). This is the highest level of cognitive processing, where agents learn (as detailed in Appendix A3). By talking with other synthetic agents and inferring their emotional and belief states, our agents learn associations between EV and beliefs *via* a high level likelihood mapping (*A*2), (updated *via* concentration parameter α). The updating of the likelihood mapping between beliefs and claims, is detailed in Appendix A7. This kind of learning is important because it provides our agents with certainty, regarding the emotional value they can expect from holding the alternative belief to the status quo, which has low precision at the beginning of the simulation (before the population is introduced to an agent proclaiming this belief).

#### 3.4.1. Perceptual learning as a coupling parameter

The learning of associations between belief and emotional valence states may be understood as a form of implicit coupling between agents (Figure 1), in that it represents an indirect and secondary influence of one agent's internal state on another. That is, sensitivity to each others' mental states is made possible only through inferences about the others' emotional state (in the absence of any overt or observable evidence for that emotional state).

In contrast to perceptual inference, learning occurs at slow time scales as mutual minimization of prediction error brings about a convergence in the parameters of hierarchical models that generate mutually sympathetic (or possibly empathetic) predictions. Parameter learning accumulates across multiple interactions, modifying generative models over a long period of time as opposed to being immediately expressed in agents' behavior. This is why perceptual learning does not bring about an immediate convergence or synchrony between interlocutors' internal states, but is only expressed in agents' adapted behavior over time.

Individuals vary in the degree to which they are sensitive to the information gained by learning associations between belief states and their potential emotional outcomes. This variation is represented in each agent's categorical probability distribution *A*2 that is updated throughout the simulation *via* a concentration parameter (α) as they accumulate information with every agent they meet. Updates to the *A*2-concentration parameters model the way in which agents' associations between belief and emotional states are based on implicit observations of others' emotional states.

The prior for this likelihood mapping is specified in terms of a Dirichlet distribution:


(16) $$\begin{array}{l} P\left(A_{sat}^{\left(2\right)}\right)=\text{Dir}\left(\alpha_{sat}^{\left(2\right)}\right) \end{array}$$

The associated approximate posterior accumulates the precision-weighted counts of correspondences between observed expressions and satisfaction levels:


(17) $$\begin{array}{l} Q\left(A_{sat}^{\left(2\right)}\right)=\text{Dir}\left(\alpha_{sat}^{\left(2\right)}+\gamma_{A}^{\left(2\right)}{\text{o}}_{expr}\ln\;{\text{x}}_{sat}^{\left(1\right)}\;\right) \end{array}$$

### 3.5. Simulated process summary for multi-agent social dynamics

**Figure 7:**
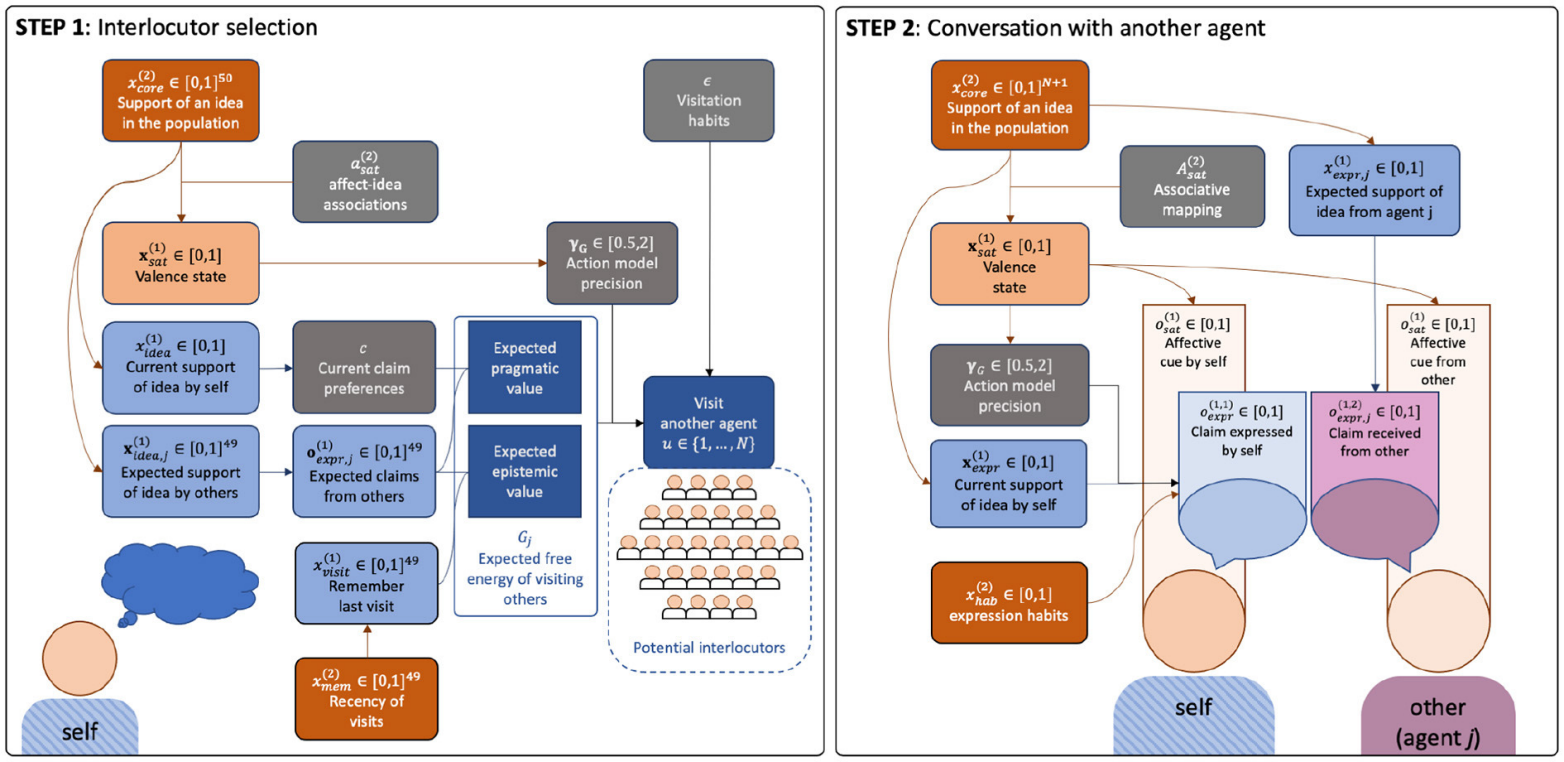
A diagram illustrating the steps of the generative model. **(Left)** Lower level Step 1: Interlocutor selection. Each day, each agent selects one interaction partner (selecting themselves means staying home). Agents cannot see each other's “opinion” before conversing. Meeting selection was conditioned on: (1) Habitual visitation drives, depending on past actions. (2) Deliberate drives, conditioned on: (2a) Expected (mis)match between expressed opinions (pragmatic value) and (2a) expected reduction in uncertainty about opinions of other agents, depending on one's memory of recent visits (epistemic value). **(Right)** Lower level Step 2: Conversation with a selected agent. Each meeting consisted in exchanges of expressed support for an idea [in the range (0,1)] and affective cues [negative-positive, in the range (0,1)]. Expressed support was conditioned on: (1) Expression habits formed during past conversations, (2) one's current support for the idea. Expressed affective cues were conditioned on one's current valence state. Affect played a role during Steps 1 and 2: Relative reliance on habitual tendencies vs. deliberation (expected free energy *G*) was regulated *via* action model precision. The latter was conditioned on one's current valence state, which was conditioned on one's current support of an idea, depending on previously learned associations between expressed ideas and concurrent affective cues (from oneself and others).

For additional clarity, we provide a verbal and graphical summary of multi-agent social dynamics in our simulations. The “community square” (depicted in Figure 6) contained 50 social agents with deep generative models (identical structure + individualized parameters), simulating how they mingled (step 1) and conversed daily (step 2) about two mutually exclusive ideas (“red” and “blue”), as illustrated in Figure 7.

#### 3.5.1. Simulated dynamics within days

Within each day, every agent engages in steps 1 and 2, generating:

- Expected support for idea (from self and others)
- Expected claim expressions (from self and others)
- Current claim preferences
- Current valence state
- Current action model precision
- Memory of most recent visit of other agents
- Current selection of an agent to visit
- Expressed opinions (when visiting and when visited)
- Expressed affective cues (when visiting and when visited)

#### 3.5.2. Simulated dynamics across days

Across days, every agent maintains (implicit) beliefs about the following:

- Support for idea from self and others
- Habits of expressed support (self)
- Recency of visits to and from others
- Visitation habits of self (dirichlet counts)
- Affect-idea associations (dirichlet counts)

## 4. Results

Active inference allows us to formulate a normative and explainable account of cultural information spread through communication by casting cultural transmission as a bi-directional communicative process that entails a particular convergence between distinct conveyors and conveners of cultural information. We provide a proof of concept for this formalization of communication dynamics by simulating a dialogue between active inference agents holding distinct beliefs and trying to convince each other of their own beliefs.

Modeling the global dynamics of a cumulative culture (i.e., the accumulation of cultural information over a manifold of transmissions), was modeled such that—at each time point all 50 agents engaged in dialogue at least once (by selecting a conversation partner).

### 4.1. Local dynamics of coupled communication

#### 4.1.1. The emergence of generalized synchrony from coupled communication

In nature, generalized synchrony emerges from sparse coupling between the internal states of dissipative chaotic systems (Pikovsky et al., 2003). In our model, generalized synchrony within a social system is operationalized as a convergence between belief states held by interlocutors (Figure 1). In other words, generalized synchrony between mutually inferring agents is understood as signaling a form of cultural reproduction of beliefs, namely, a mechanism by which previously distinct internal states merge and combine into one. This convergence is made possible through a particular coupling between the internal states of cultural entities, under which generalized synchrony is an emergent phenomenon. We hypothesized that without active perception and mutual model updating, belief convergence would be precluded, since interlocutors' inner states would be inaccessible to each other. That is, agents' ability to actively infer hidden states in the world and update their own model according to the sensory evidence they receive is the foundation for achieving generalized synchrony in a social system.

**Figure 8:**
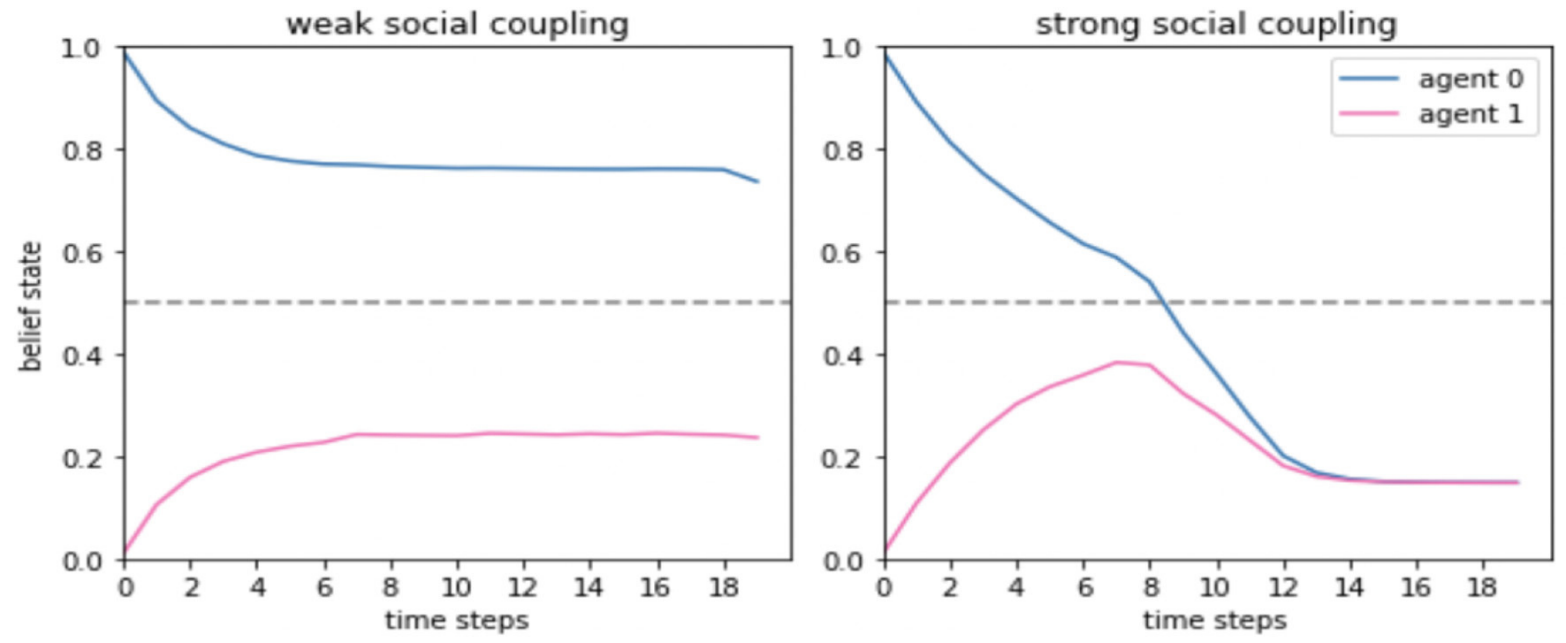
Sensitivity to observable evidence modulates the level of social coupling between agents in dialogue. This Figure and its legend were adapted from an open-source preprint of a conference paper, with permission of the authors (Figure 2 of Kastel and Hesp, 2021). In our simulations, communicated cultural information takes the form of an internal belief state held by agents with a certain probability described under the A1 matrix (Figure 3A). While this internal state is defined as a binary variable, an agent's beliefs are given by a categorical probability distribution that can take on any real number in the range (0,1). This figure shows the belief states (vertical axes) of two agents (represented in blue and pink) as they engage in dialogue across 18 time steps (horizontal axis). When the likelihood precision is low for both agents **(Left)** their internal states are very weakly coupled, such that each agent sticks to their own belief and does not attune to the claims of the other. In contrast, when both agents have high precisions **(Right)** their engagement in mutual attunement is facilitated and their beliefs converge onto one shared belief, which is then installed in both of their generative models as a shared narrative.

Our results indicate that agents' ability to listen and attune to the claims of their partner is indeed limited to the extent that they are sensitive to sensory evidence from their encultured environment (Figure 8).

To understand the implications of these findings, it is important to shed light on the way they tie in to previous work on active inference communication. In Friston and Frith (2015) provided evidence for the notion that generalized synchrony becomes altogether unattainable when agents do not possess sufficiently similar generative models. Our results go beyond this and provide evidence for the idea that only when generalized synchrony is attainable (i.e., when interlocutors possess sufficiently similar generative models), communication underlies a convergence between agents' belief states. Our simulations should therefore be understood as taking generalized synchrony for granted while providing evidence for the premise that the level to which agents' beliefs converge (i.e., the level of synchrony between their internal states) is modulated by their sensitivity to model evidence (*A*1).

### 4.2. Global dynamics of cumulative culture

**Figure 9:**
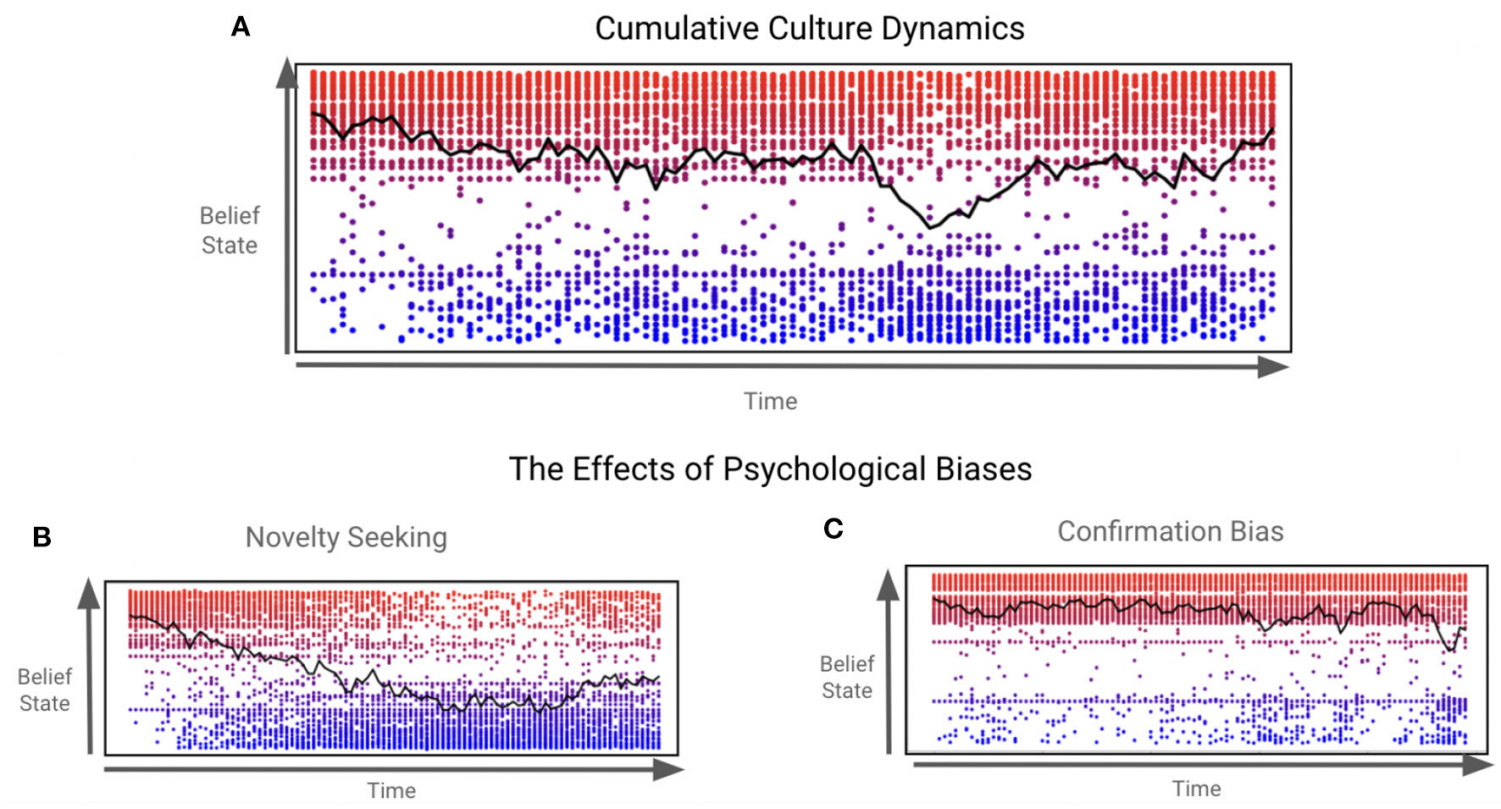
The emergence of cumulative culture. This Figure and its caption were adapted from an open-source preprint of a conference paper, with permission of the authors (Figure 3 of Kastel and Hesp, 2021). These plots depict the evolution of population-averaged support (black line) with regards to the idea that represents the initial status quo (top indicates 100% support, bottom indicates 0% support) over time (horizontal axes) along with individual core beliefs (shown in the underlying scatter plot, explained below) in three simulations, for which only the relative size of the subgroup with high confirmation bias was modulated [**(A)**: 5%, **(B)**: 15%; **(C)**: 85%]. The underlying scatter plots indicate the core beliefs of individual agents by means of their vertical location as well, with a color scale for additional clarity (red indicating maximal support for the status quo, blue maximal support for the novel idea). **(A)** Simulation of a Cumulative Culture: In this reference simulation, half of all agents are parameterized with high confirmation bias. When a divergent belief state (blue) is introduced to the status quo population (red) at the first time step, it spreads through it *via* agents in dialogue that cumulatively change the belief structure within the population. Notably, the introduction of a divergent belief seems to split the population into two subgroups: those supporting the new idea, and those adhering to the previous status quo. This effect is modulated by agents' individual strategies for choosing which interlocutors to engage with (s3). **(B)** High levels of novelty seeking in the population: When only 15% of agents are parameterized with high confirmation bias, the population exhibits high levels of novelty seeking and ends up being divided in favor of the divergent belief state, with more agents eventually holding this belief than the status quo. **(C)** High levels of confirmation bias in the population. When 85% of agents are parameterized with high confirmation bias, the population is divided in favor of the status quo belief, with more agents holding to this belief than the new and divergent belief.

Our simulations of a cumulative culture should be understood as modeling the dynamics of a culture that is the sum (or accumulation) of modifications to cultural beliefs and practices over time (Figure 9). While the local dyadic dynamics simulated in the previous section illustrate convergence to shared belief states held by individual agents, our global simulations leverage this synchronization to evince emergent dynamics within the population. We now review the key (predicted and) emergent phenomena we observed under this model of cumulative culture:

#### 4.2.1. The introduction and spread of a novel belief induces segregation within a population

When a divergent (non status quo) belief state propagates within our synthetic population, it brings about segregation into sub-groups. Qualitatively, this is represented as a visible separation between two groups of agents: those that hold a belief that approximates the status quo (presented in red), and those that approximate the alternative, divergent belief (presented in blue).

In active inference, this communicative isolation (where agents gradually form groups of individuals they would prefer to converse with) can be explained by the attunement of interlocutor's generative models on the microscale, which translates over time—and with multiple encounters—into collective free energy minimization on the macroscale. On the microscale, local efforts to minimize free energy are expressed as agents' disinclination for meeting interlocutors that hold intractably divergent beliefs. On the macroscale, these local efforts translate into a global collective behavior of self organized separation between incongruent groups of agents, such that communicative isolation best ensures both local and collective free energy minimization. In other words, when an intractable divergent belief propagates within a homogenous population, communicative isolation between incongruent groups emerges as a strategy to minimize expected free energy, while the same strategy homogenizes the belief states of agents within congruent groups. It is interesting to reflect on the observation that the size of the two groups was roughly equivalent; a phenomena that characterizes many instances of cultural convergence (Myerson and Weber, 1993); e.g., the voting in the UK for Brexit.

#### 4.2.2. Local psychological biases modulate population level segregation

The above simulations also show how differences in parameters that determine levels of confirmation bias (manipulated directly through $A_{C}^{\left(2\right)}$; see Figure 5) and novelty seeking (emerging from *G*) affect the segregation within the population into groups of agents holding either congruent beliefs or the alternative belief. When confirmation bias is relatively low (Figure 9B), the population evolves such that the majority of agents end up subscribing to the alternative belief. However, when confirmation bias is relatively high (Figure 9C), the majority of agents remains convinced of the previous status quo.

These results indicate that confirmation bias suppresses tendencies of the population as a whole toward the adoption of an idea that diverges from the status quo. When the confirmation-driven fraction of the population is relatively low (15%), we naturally observe more novelty-seeking behaviors, indicating agents are more “open-minded” and willing to meet with agents of unknown beliefs. They are intrinsically encouraged by their own curiosity to expose themselves to novel expressions. Once such agents become convinced by such interactions, they can start to popularize it for the rest of the population. If the population is, however, made up of a majority of agents driven by confirmation bias, they do not engage as much with the alternative belief and popularization is precluded.

These results are reminiscent of the widely used “adopter categories” theory, a theoretical framework outlined by Everett Rogers in his book “Diffusion of Innovations” which defines five groups in terms of their relative precession in adopting an innovation (Rogers et al., 2014). According to this framework, the first two groups to adopt an innovation are innovators and early adopters, which make up 2.5 and 13.5% of the population, respectively (Sahin, 2006). Our results appear to be consonant with the finding that relatively small numbers of early adopters and innovators play a significant role in the propagation of an innovation to other segments of the population (Dedehayir et al., 2017). One explanation for this phenomenon is that innovators and early adopters communicate innovations and their relative advantages to other segments of the population, thereby popularizing them.

## 5. Discussion

In this paper, we provide an active inference framework for the emergence of a cumulative culture from joint communication dynamics. The principal achievement of this framework is that it offers an overarching, quantitative and multiscale account against which multiple hypotheses from different domains of the social sciences may be universally tested. This accomplishment has the potentiality to bring the replication crisis faced by the psychological and social sciences in the past decade, a step closer to a resolution. A formal, standardized model of cultural evolution can evoke such an outcome as personal intuitions and culturally biased folk theories that currently make results difficult or impossible to reproduce, will become anchored to an objective and universally agreed upon verifiable account.

Notably, our framework offers a multiscale approach to the understanding of cultural evolution processes, as expressed in at least three ways. Firstly, it refers to the intrinsically hierarchical nature of the generative models themselves characterizing affective, cognitive, and behavioral dynamics (described at length in the text and summarized in Figure 2). Secondly, we combined this with the hierarchical nature of the dyadic interaction process (described at length in the text and summarized in Figure 7). Thirdly, these dyadic interactions were contextualized by a multi-agent setting for which parametrizations themselves were generated procedurally from population-level distributions of hyperparameters (described at length in the text and Appendix, illustrated in Figure 6) and including two subgroups (described in Figure 5). Our simulations depict cultural dynamics that arise from one another to form nested levels of hierarchical organization, quintessential to complex dynamical systems. This novel way of modeling cultural dynamics across layers of organization accord nicely with new approaches to artificial intelligence that originate from the notion that intelligence emerges as much from cells and societies as it does from individuals. The emerging field of biologically inspired artificial intelligence involves computational approaches that model biological systems on various layers of organization. Such artificial intelligence systems include: cellular systems; neural systems; immune systems; bio-mimetic, epi-genetic and evolutionary robots as well as collective systems. In this section we will discuss the specific implications of our multilevel cultural simulations on the field of biologically inspired artificial intelligence.

### 5.1. Communication models for biologically inspired artificial intelligence

Traditionally, AI has been concerned with representing the behaviors and architectures of human cognition. The preoccupation with human intelligence stems from the widely accepted notion that despite being neither the strongest nor the fastest species on earth, humans occupy a distinctly dominant position. Intellectual in nature, this dominance has previously been attributed to our culture, morality and language. However, in most of these social-cultural capacities, great apes share striking similarities with humans, yet still do not show human level intelligence, which leaves social scientists wondering about the underlying roots and causes of human intelligence. Recent studies show that despite their striking similarity to humans in most social-cultural domains, great apes are not cognitively equipped for the kinds of social coordination with others that is evident in humans (Krupenye et al., 2016; Tomasello, 2018). These findings suggest that humans might owe their remarkable intelligence to their unique ability to coordinate their behavior through joint communication and other (non verbal) cultural exchanges.

The idea that humans' cognitive skills are the result of shared intentionality, coordination, communication and social learning is known as the ontogenetic adaptation hypothesis (Tomasello, 2020). This theory stipulates that animals use social learning to gather information from their conspecifics about challenges in their environment while avoiding some of the energetic and time costs associated with a-social, trial and error learning (Clark and Dumas, 2016). According to this, social interactions- and specifically, communication and coordination- are a crucial component of human intelligence.

This makes a strong case for the use of communication models as inspiration for the development of socially intelligent artificial agents. Indeed, equipping artificial agents with the ability to accurately coordinate and communicate with other agents in their environment may well be a crucial missing piece in the modeling of advanced- human level- cognitive abilities. By modeling the underlying dynamics of social communication and coordination as we have in this paper, we bring to light an otherwise unexplored topic, which may be one of the most promising directions for achieving human level machine intelligence.

### 5.2. Cumulative culture models for biologically inspired artificial intelligence

The social sciences are on the verge of a revolution, where researchers begin to have a more complex understanding of the ways in which cultural practices and social choices interplay with, and shape human experiences. Specifically, it is becoming clear that individual intelligence is not what makes us- above other species- uniquely intelligent. Rather, the last decade has brought with it the notion that the cumulative nature of human culture is responsible for our exceptional cognitive capabilities and intelligence as a species. This capacity to acquire- across generations- highly evolved and complex social systems such as language, cities and technologies, are said to have sharpened humans' cognitive capacities and survival strategies in a way that no other species has ever had the privilege to experience (Henrich, 2015).

Other related theories suggest that individuals are not “the brains” behind a creative idea, but that innovation is in fact a product of a collective cultural brain (Muthukrishna and Henrich, 2016). According to this, The ideas of individuals do not stand in competition or comparison with other agents in the population, but are better understood as a nexus for previously isolated ideas within it. This collective approach to cultural innovation is supported by empirical findings showing that innovation rates are higher in cultures with high sociality (i.e., large and highly interconnected populations that offer exposure to more ideas), transmission fidelity (i.e., better learning between agents) and transmission variance (i.e., a willingness to somewhat deviate from the accepted learned norms) (Muthukrishna and Henrich, 2016).

Although the capacity for cumulative culture (i.e., the capacity to acquire complex social systems through learning that accumulates across generations) in animals remains contentious (Dean et al., 2014), their expression of collective intelligent systems in swarms (Chakraborty and Kar, 2017), ant-colonies (Blum, 2005), flocks of birds (Boucherie et al., 2019), schools of fish (Boucherie et al., 2019) and other social systems, is evident in nature and has become an integral part in the field of artificial intelligence as more and more high complexity problems require bio-inspired solutions that are achievable within a reasonable period of time.

To the extent that the cumulative and collective nature of culture provides an accurate account of intelligence, as theories suggest, investigating the underlying mechanisms of intelligence may be informed by the investigation of complex social-cultural systems. In this case, providing a quantitative and measurable account of the way a “collective brain” emerges from simple, local rules of operation (namely, joint communication), as we have illustrated in this paper, becomes invaluable in the pursuit of machine intelligence.

### 5.3. Embodied active inference for biologically inspired artificial intelligence

Embodied cognition is the theory that many elements of cognition are shaped by elements of the entire body of the organism. While emphasizing the circular causality between the environment and the individual, social embodiment suggests that embodiment in social beings plays a significant role and improves upon social interactions. Justifications for social embodiment are that different body states (such as postures and facial expressions) enhance the communicative skills of embodied agents and consequently, play a central role in social information processing such that interactions between embodied agents and humans are facilitated (Bolotta and Dumas, 2021).

A natural speculation may be that robots have better skills of communication and inter-robot social inference and expression than digital avatars, since they can use their bodies for behavioral expression and coordination with other robots. However, we argue that despite lacking a physical body, active inference avatars are embodied in that the computational formalism that is applied to them (namely, active inference) implies embodiment. Technically, what this means is that active inference agents in our simulations adhere to three formal conditions for having embodied cognition:

1. They have a perceptual system which allows them to gather culturally relevant information from their surroundings. This is evident in the first layer (“perception”) of the hierarchical structure of the generative model of the agents (Figure 2).
2. They have a motor system that allows them to communicate their internal states to their social environment. This is evident in the third layer (“action”) of the hierarchical structure of the generative model of the agents (Figure 2).
3. They are situated in their environment such that they are able to manipulate their dynamic surroundings through their actions. This is evident in agents' ability to listen and attune to each others' belief expression in a way that allows for a coupling of their internal states and the emergence of generalized synchrony between them (Figure 8).

The fact that agents under the active inference formulation conform to these three conditions is non trivial, and it points to the fact that these agents could not be simply replaced by any hypothetical- non embodied- simulated intelligent being. In other words, our simulations would not make sense unless applied to a population that adheres to the certain criteria aforementioned. We could only apply our simulations to agents that adhere to all these conditions (i.e., have embodied cognition), or our simulations simply would not work. In this sense, our agents may not be physically embodied robots, but we argue that- by definition- as active inference agents capable of perceptions and actions in a situated environment, they are software embodied agents. Had we put this software into social robots that had the hardware equivalent of “ears” and “mouths,” we would be able to produce embodied robots in a way that would improve their social interactions. Crucially, we argue here that embodiment must be present in both the software and the hardware for social interactions of agents to be enhanced by it, and that the active inference formalism implies embodiment for the former.

### 5.4. Limitations and future research

Although this paper provides important insights into the underlying dynamics of social-cultural systems, it entails certain limitations that will now be outlined and may be addressed in future research.

First, our communication simulations assume that social exchange is limited to a dyad, when in fact generalized synchrony in nature may occur between multiple coupled systems. Our formulations of the exchange of social information as communication therefore represent only a specific case of generalized synchrony that might highlight a much more encompassing phenomenon. As an example, through social media, cultural information can reach large populations at a given time point. The idea that generalized synchrony between inferring agents may go beyond the emergent behavior of two communicators and exist between ensembles of coupled self organizing systems has also been considered in the active inference literature (Palacios et al., 2019).

Second, while we provide a formulation of the way modifications to cultural information occur during communication (i.e., the transmission of social information) and we have simulated the emergence of cumulative culture from these dynamics (i.e., the prevalence of social information), we have not provided an account of the way novel social information is introduced into a population to begin with. We have assumed that belief states are gradually modified with every cultural exchange, such that the outcome of this exchange may be considered novel by virtue of it being a unique recombination of existing beliefs and practices. Future research may focus on asking important questions like: Why are we inclined to say that innovation is a unique event that does not occur with every cultural transmission? More importantly, how can we define and even model the difference between a slight modification to a cultural trait and innovation?

**Figure 10:**
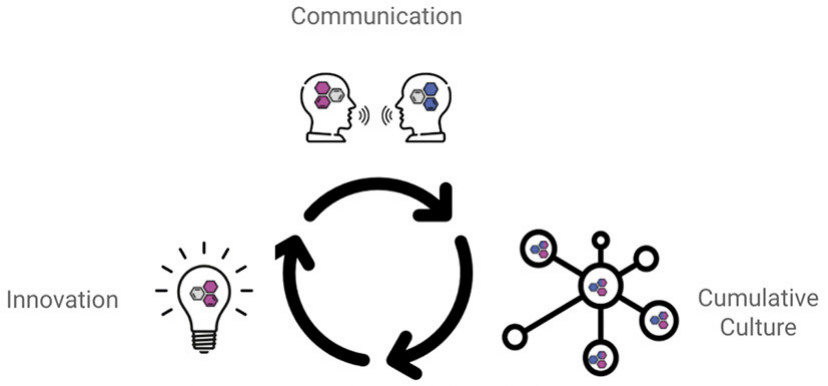
The circular dynamics of cultural evolution.

The importance of identifying exactly what constitutes innovation and how to model its emergence is critical for an accurate understanding of socio-cultural dynamics because it would bring the circular dynamics of a complex culture to a required close (Figure 10). Under such an account, not only would cumulative culture naturally emerge from a complex network of agents engaged in joint communication (as shown in this proposal), but innovation would emerge from cumulative culture and underlie communication in a repeating, recursive loop that is the hallmark of complex dynamical systems.

In the simulation environment presented here, efficient communication could be considered, to some extent, as reflecting local communicative needs and these needs are grounded in properties of this socio-cultural environment. However, the environment itself does not impose any of the practical constraints known to drive real-life human behaviors (e.g., need for food, warmth, hygiene). Previously, researchers have argued that practical benefits in adapting to the environment tend to accelerate the repetition and widespread adoption of cultural practices (Kashima et al., 2019). In future work, the authors aim to expand on these notions by enriching the simulated environment with actual practical constraints.

In the current work, emotional valence was tied to action confidence in a top-down manner (it affected action-model precision for both meeting selection and expression) but not in a bottom-up manner (e.g., based on action outcomes). For the sake of simplicity, influences on emotional valence were purely associative (i.e., based on emotional expressions of conversation partners). Therefore, one natural and valuable extension of these simulations in future work would be to fully incorporate the recursive and principled formulation of emotional valence that has been derived from deep active inference (Hesp et al., 2021), which naturally tracks changes in subjective fitness. This recurrent formulation, in particular when combined with imagination-induced affect (see Hesp et al., 2020), will specifically benefit from the grounding of these simulated cultural exchanges in a more elaborate virtual environment combined with agents that have actual bodily and social needs such that subjective fitness estimates (based on action-model precision) come to confer some practical relevance (as described in the preceding paragraph).

Finally, another limitation of our simulations is that the agents' freedom for choosing the interlocutors they want to engage with, might bias cultural transmission in a way that does not apply to some forms of social interaction. Specifically, meeting selection, or the freedom to voluntarily select the transmitting interlocutor, does not extend to social interactions in which agents do not have a choice in determining the source of their cultural learning. For example, during development, children are constantly exposed to individuals, social situations, cultural practices, and conversations that they do not voluntarily select. In this case, it is the Parents' culturally dominant behaviors that play a central role in the development of children's internalization of cultural beliefs, rather than the voluntary actions of their children (Fernald and Morikawa, 1993; Senzaki et al., 2016).

## 6. Conclusion

In this paper, we employed a Bayesian framework—known as active inference—to formally account for the dynamics underlying (local) communication and (global) cumulative culture dynamics, thus contributing to the ever-growing body of research on multi-agent Bayesian models (e.g., Gunji et al., 2018) and collective active inference (e.g., Friedman et al., 2021; Heins et al., 2022) Under our account, the social “transmission” of cultural information has been cast as a fundamentally bidirectional process of communication, which has been shown in the previous active inference literature to induce a generalized synchrony between the internal (belief) states of agents holding sufficiently similar generative models. Building on this work, we operationalized generalized synchrony as a particular convergence between the internal states of interlocutors, and show that it depends sensitively on the precision of observation or likelihood mappings in a generative model of communicative exchange. When we simulate a population of agents that simultaneously engage in communication over time, cumulative culture emerges as the collective behavior brought about by local belief updating (active inference and learning in a dyadic setting). Our simulations show that when a divergent belief is introduced to the status quo, it spreads within the population and brings about a collective behavior characterized by a certain degree of segregation between different belief groups. The level to which the status quo population defects to the divergent belief is mediated by local psychological biases for confirmation bias (as directly manipulated) and novelty seeking (as emergent from procedural generation of parameters). These cultural (c.f., voting) equilibria are minimizers of collective or joint free energy that emerge from the imperative to minimize uncertainty and surprise in dyadic exchanges.

## Data availability statement

The original contributions presented in the study are included in the article/Supplementary material, further inquiries can be directed to the corresponding author.

## Author contributions

NK and CH implemented the formalism of active inference, conducted the simulations, and made the figures. NK developed the theoretical background for the manuscript and wrote up the first draft of manuscript. KR and CH edited the manuscript and linked components in the literature review with prior work in the field. CH and KF edited the manuscript and further developed the formalism of active inference in the methods, generative model, and results sections. All authors contributed to the article and approved the submitted version.
