# Corrigendum: Small steps for mankind: Modeling the emergence of cumulative culture from joint active inference communication

**DOI:** 10.3389/fnbot.2023.1151062

**Published:** 2023-02-08

**Authors:** Natalie Kastel, Casper Hesp, K. Richard Ridderinkhof, Karl J. Friston

**Affiliations:** ^1^Amsterdam Brain and Cognition Centre, University of Amsterdam, Amsterdam, Netherlands; ^2^Institute for Advanced Study, University of Amsterdam, Amsterdam, Netherlands; ^3^Precision Psychiatry and Social Physiology Laboratory, Department of Psychiatry, CHU Sainte-Justine Research Center, Université de Montreal, Montreal, QC, Canada; ^4^Wellcome Centre for Human Neuroimaging, University College London, London, United Kingdom; ^5^Department of Developmental Psychology, University of Amsterdam, Amsterdam, Netherlands

**Keywords:** active inference, generalized synchrony, communication, social dynamics, cumulative culture, complex systems

In the published article, there was an error in the Funding statement with regards to the omission of the Wellcome Trust Principal Research Fellowship (KF; Ref. 088130/Z/09/Z). The original text was: “This research was undertaken thanks in part to funding from a NWO Research Talent Grant of the Dutch Government (CH; No. 406.18.535).” The correct Funding statement appears below.

