# Supplementary material for: Small steps for mankind: Modeling the emergence of cumulative culture from joint active inference communication
Source: Front Neurorobot. 2023 Jan 9;16:944986. doi: 10.3389/fnbot.2022.944986 (PMC9868743; doi:10.3389/fnbot.2022.944986)
Supplement: Supplementary file 1 [file Data_Sheet_1.PDF]

-Appendices-

These appendices have been reprinted with permission from the authors from an open-source preprint publicly available on Research Gate.

Appendix A1. HIGHER-LEVEL HIDDEN STATE FACTORS:

$x^{(2)}: \{x^{(2)}_{core},$ *[core beliefs of self and others about a particular claim, across days]*

$x^{(2)}_{mem},$ *[memory of having visited each agent]*

$x^{(2)}_{habit}\ \}$ *[habits of self, across days]*

Appendix A2. LOWER-LEVEL HIDDEN STATE FACTORS (specify events on a given 'day')

$x^{(1)}: \{\ x^{(1)}_{loc},$ *[self location, where each agent has a unique 'home' location]*

$x^{(1)}_{belief},$ *[beliefs of self and others about a particular claim]*

$x^{(1)}_{visit},$ *[beliefs about having visited each agent]*

$x^{(1)}_{sat}\quad \}$ *[satisfaction of self and others]*

Appendix A3. HIGHER-LEVEL GENERATIVE MODEL:

$x^{(1)}_{belief} = A^{(2)}_{belief} x^{(2)}_{core}$ *[core beliefs specify prior expectations for beliefs on the lower level]*

$x^{(1)}_{sat} = A^{(2)}_{sat} x^{(2)}_{core}$ *[core beliefs specify satisfaction states for the lower level]*

$x^{(1)}_{visit} = A^{(2)}_{mem} x^{(2)}_{mem}$ *[memory specifies beliefs about having visited each agent on the lower level]*

$E_{expr} = A^{(2)}_{expr} x^{(2)}_{habit}$ *[habits of self specify prior tendency for belief expression]*

$x^{(2)}_{T+1} = B^{(2)} x^{(2)}_{T}$ *[higher-level states decay over time: gradual forgetting]*

Appendix A4. LOWER-LEVEL GENERATIVE MODEL FOR ACTION:

Action model for meeting selection:

In our simulations, we have incorporated psychological biases in agents' preferences for meeting similar (i.e., belief compatible) or unknown agents. Note that while agents biased toward confirming beliefs would tend toward individuals with similar beliefs to their own, novelty seekers would not look for the opposite of this (i.e., look for individuals with divergent beliefs to their own), but rather prefer individuals of yet unknown beliefs.

In active inference, action selection is guided by the expected free energy [G], which entails maximizing the expected utility of an action (known as pragmatic value), while also maximizing the expected information gain afforded by future actions – by reducing uncertainty about the causes of outcomes (known as epistemic value). These constraints on action selection could be interpreted as formalizing the exploration-exploitation trade-off in Bayes-optimal systems. Active-inference agents therefore maximize epistemic value until information gain is low, after which the maximization of pragmatic value and exploitation are assured (Friston, Rigoli, Ognibene, Mathys, Fitzgerald & Pezzulo, 2015).

In our model, the agents' choice of interlocutors with known and similar beliefs versus those with unknown beliefs can be cast in terms of a tradeoff between pragmatic and epistemic value. On the one hand, a confirmation bias emerges from the maximization of expected utility, increasing synchronization between interlocutors' internal models, thus allowing for the emergence of shared expectations (Hesp et al., 2019). On the other hand, novelty seeking emerges from the maximization of information gain, allowing for the exploration of the sample space. Also understood as intrinsically motivated curious behavior (Friston, Lin, Frith, Pezzulo, Hobson & Ondobaka, 2017), maximization of epistemic value allows individuals to better predict the consequences of their actions (e.g., which agent to meet) through greater certainty about the hidden states of their environment (e.g., the beliefs of other agents).

From the point of view of agents in our simulations, increasing pragmatic value translates into selecting interlocutors with similar beliefs, while increasing epistemic value translates into selecting agents whose beliefs are unknown or highly uncertain (this way, a meeting increases information gain). From this point of view, it is clear the two imperatives constrain each other and maximizing both simultaneously is partially (but not entirely) paradoxical. While maximizing pragmatic value requires agents to choose an interlocutor they know is similar to them, maximizing epistemic value necessitates they meet with a stranger they do not know at all.

$$P(u_{\text{visit}}) = \sigma\left(-\boldsymbol{\gamma}_{G,\text{visit}}\mathbf{G}_{\text{visit}} + \gamma_{E,\text{visit}}\mathbf{E}_{\text{visit}}\right)$$
$$\mathbf{G}_{\text{visit}} = \mathbf{o}_{\text{expr,visit}} \cdot (ln\ \ \mathbf{o}_{\text{expr,visit}} - \mathbf{C}_{\text{idea}})\ + \mathbf{H} \cdot \mathbf{x}_{\text{idea},visit}$$
$$x^{(1)}_{\text{idea,visit}} = \mathbf{B}^{(1)}_{\text{visit}} x^{(1)}_{\text{idea,home}}$$
$$\mathbf{o}_{\text{expr,idea}} = \mathbf{A}^{(1)}_{\text{idea}} \mathbf{x}^{(1)}_{\text{idea,visit}}$$
$$\mathbf{C}_{\text{idea}} = ln\left(\mathbf{A}^{(2)}_{C}\mathbf{x}^{(2)}_{core}\right)$$

$if\ x_{visit,j} = 1$: [equals 1 if agent recently visited a particular agent *j*]

  $H_j = 0$ [ambiguity is zero if agent visited this agent *j* already]

$else$:

  $H_j = 0.1$ [ambiguity is non-zero if agent has not visited agent *j* yet]

Appendix A5. ACTION MODEL FOR BELIEF EXPRESSION OF EACH AGENT

$$\mathbf{x}^{(1)}_{sat} = \mathbf{A}^{(2)}_{sat}\mathbf{x}^{(2)}_{core}$$
$$\beta_{expr} = \beta^{(+,-)} \cdot \mathbf{x}^{(1)}_{sat},\ \beta^{(+,-)} = [0.25, 2.0]$$
$$P(\gamma_{expr}) \approx \Gamma(1, \beta_{expr})$$
$$\boldsymbol{\gamma}_{expr} = E_P[\gamma_{expr}] = 1/\beta_{expr}$$
$$P(u_{expr}|\gamma_{expr}) = \sigma\left(-\boldsymbol{\gamma}_{expr}\ ln\ \ \mathbf{x}^{(2)}_{core}\ + \gamma_{E,expr}\mathbf{E}_{expr}\right) = \mathbf{u}_{expr}$$

Appendix A6. GENERATIVE PROCESS:

Generative process for meeting selection:
$u_{loc} \sim P(u_{loc})$ *[actual meeting* u_{loc} *is sampled from meeting selection prior* $P(u_{loc})$*]*

Generative process for belief expression of each agent:

At this level of cognitive control, agents call on a series of constraints underlying the selection of a particular belief for expression (u2). Beyond the low level habitual factor [E], this action involves multiple higher order considerations. First, an agent considers their core belief state (x), and the way this state a priori maps on to one of two discrete emotional valence states (s2) via an initial likelihood mapping [A2] Emotional Valence (EV) is defined as the extent to which an emotion is positive or negative (Feldman Barrett & Russell, 1999), such that agents' core beliefs are a priori associated with either positive emotional valence or negative emotional valence (with some probability). As a minimal form of vicarious learning, the initial mapping is further updated based on associations the agents observe between their interlocutors' expressed belief state and EV value. The initial mapping therefore involves minimal precision for the expected EV for an incongruent belief since agents are first introduced to this belief (and associated EV) during the simulations. For this reason, the initial likelihood mapping between states is updated throughout our simulation via a concentration parameter (α).

EV states are generated from core belief states, using a (learnable) likelihood mapping:

$$\mathbf{x}_{sat}^{(1)} = \mathbf{A}_{sat}^{(2)}\mathbf{x}_{core}^{(2)}$$

Confidence in belief expression is generated using a Gamma distribution, where the rate parameter expresses the Bayesian model average of (+,-) values associated with high and low satisfaction:

$$\beta_{expr} = \beta^{(+,-)} \cdot \mathbf{x}_{sat}^{(1)},\ \beta^{(+,-)} = [0.25,2.0]$$

$$P(\gamma_{expr}) \approx \Gamma(1, \beta_{expr})$$

The expression of beliefs is guided by current core beliefs (scaled with satisfaction-dependent expr) and by habitual belief expression Eexpr(scaled with a fixed parameter E,expr):

$$P(u_{expr}|\gamma_{expr}) = \sigma\left(-\gamma_{expr}\ ln\ \mathbf{x}_{core}^{(2)} + \gamma_{E,expr}\mathbf{E}_{expr}\right) = \mathbf{u}_{expr}$$

The intrinsically stochastic and itinerant nature of the generative process of communication is modeled by using a two-dimensional Dirichlet distribution to generate observed expressions in the range [0,1], where each agent's belief expression prior $P(u_{expr}|\gamma_{expr})$ is used to specify their concentration parameters (multiplied by 12 to reduce variance):

$$\mathbf{o}_{expr} \sim Dir(12\mathbf{u}_{expr})$$

Generative process for emotional valence expressed by each agent:

$\mathbf{o}_{sat} = \mathbf{A}^{(1)}_{sat}\mathbf{x}^{(1)}_{sat}$ *[satisfaction observed by interaction partner corresponds to actual satisfaction]*

The EV state predicted is then used to generate an action confidence value (γ) such that positive EV generates high confidence in a certain expression of the belief state (u1) and negative EV generates low confidence values. Higher confidence values assign higher precision to the expected free energy (G) for the expression of beliefs in the current conversation.

Appendix A7. PERCEPTION.

Updating beliefs about the other agent's belief based on their expression:

$$Q\left(x^{(1)}_{belief}\right) = \mathbf{o}_{expr}$$

Updating of core belief based on beliefs expressed by other agents:

$$Q\left(x^{(2)}_{core}\right) = \sigma\left(ln\, x^{(2)}_{core} + \gamma^{(2)}_{A,self} ln\, \mathbf{o}_{expr,self} + \gamma^{(2)}_{A,other} ln\, \mathbf{o}_{expr,other}\right)$$

A8. LEARNING.

Habit learning for meeting selection:

$$P(E_{loc}) = Dir(e_{loc})$$
$$Q(E_{loc}) = Dir(e_{loc} + 0.05u_{loc})$$

Habit learning for belief expression:

$$P\left(E_{expr}\right) = Dir\left(e_{expr}\right)$$
$$Q\left(E_{expr}\right) = Dir\left(e_{expr} + 0.1o_{expr}\right)$$

Perceptual learning for the mapping between satisfaction and core beliefs, based on the expressions of other agents:

$$P\left(A^{(2)}_{sat}\right) = Dir\left(a^{(2)}_{sat}\right)$$
$$Q\left(A^{(2)}_{sat}\right) = Dir\left(a^{(2)}_{sat} + \gamma^{(2)}_{A} o_{expr}\, ln\, ln\, x^{(1)}_{sat}\right)$$

Appendix A9. INITIALISATION OF PARAMETERS FOR EACH AGENT.

We ensure a diversity of generative models utilized within the population by allocating different initial values for the precision parameters (A, B, C, E) of each agent, randomly selected from a gamma distribution. A detailed account of the initialization of parameters for each agent is provided below.

While G (expected free energy) does not have an initial value per se (as expected free energy across policies emerges from the interplay of other precision parameter values), our simulation incorporates a confidence parameter (γG) that regulates the impact of G on action selection and is initialized for every agent via a random selection from a gamma distribution.

This has implications for the parameterization of psychological biases toward exploration vs. exploitation that are also incorporated in our model. While the degree to which agents are biased towards conforming their own beliefs [C] is initialized from a gamma distribution, the level to which they incorporate a bias toward novelty is driven by emergent values of expected free energy over policies [G].

Distinct belief states for agents in our simulation are also initialized, such that all but one agent range in the proximity (with slight variability between agents) of belief 1, the status quo, at time step 0. At time step 1, an agent holding belief 0 is introduced to the simulation. This agent introduces the divergent belief state to the population, where it then propagates through dyads of communication. A unique level of satisfaction (from the held belief state) for each agent is also initialized by sampling from a gamma distribution.

$\gamma^{(2)}_{A,belief} \sim \Gamma(5{,}6)$ *[regulates the integration of beliefs of other agents in one's own core belief]*

$\gamma^{(2)}_{A,sat} \sim \Gamma(10{,}1)$ *[regulates learning rate of mappings between satisfaction and core belief, based on observed correspondences in other agents]*

$\gamma_{G,loc} \sim \Gamma(1{,}1)$ *[regulates reliance on action model in selecting agent to meet]*

$\gamma_{E,loc} \sim \Gamma(1{,}1)$ *[regulates reliance on habitual prior in selecting agent to meet]*

$\gamma_{E,expr} \sim N\left(\frac{\gamma_{E,loc}}{10}, \frac{\gamma_{E,loc}}{200}\right)$ *[regulates reliance on habitual prior in expressing action, which correlates with $\gamma_{E,loc}$]*

$\gamma^{(2)}_{B,core} \sim \Gamma(4, .5)$ *[regulates stability of core beliefs across days]*

$\gamma^{(2)}_{B,habits} \sim \Gamma(.5{,}1)$ *[regulates stability of expression habits across days]*

$B^{(2)}_0 = \big[[.75, .25], [.25, .75]\big]$ *[specifies baseline transition probabilities]*

$B^{(2)} = \sigma\left(\gamma^{(2)}_B \; ln \;\; B^{(2)}_0\right)$ *[corrects $B^{(2)}_0$ using the agent-specific $\gamma^{(2)}_B$ values]*

Agents with relatively weak confirmation bias:

$A^{(2)}_C \sim Dir(6{,}4)$ *[induces weak reliance on core beliefs for specifying lower-level preferences]*

Agents with relatively strong confirmation bias:

$A^{(2)}_{C,1} \sim Dir(999{,}1)$ *[induces strong reliance on core beliefs for specifying lower-level preferences]*
